# ATR safeguards epithelial-to-mesenchymal transition by countering R-loops and enabling transcription reprogramming

**DOI:** 10.1172/JCI192225

**Published:** 2026-01-22

**Authors:** Parasvi S. Patel, Jacob P. Matson, Xiaojuan Ran, Marcello Stanzione, Ajinkya S. Kawale, Mingchao Wang, Sneha Saxena, Conrad Sander, Jacquelyn Curtis, Jessica L. Hopkins, Edmond Wong, Ryan B. Corcoran, Daniel A. Haber, Nicholas J. Dyson, Shyamala Maheswaran, Lee Zou

**Affiliations:** 1 Massachusetts General Hospital Cancer Center, Harvard Medical School, Charlestown, Massachusetts, USA.; 2 Department of Pharmacology and Cancer Biology, Duke University School of Medicine, Durham, North Carolina, USA.; 3 Howard Hughes Medical Institute, Massachusetts General Hospital, Charlestown, Massachusetts, USA.

**Keywords:** Cell biology, Oncology, Cancer

## Abstract

Transitions of cancer cells between distinct cell states, which are typically driven by transcription reprogramming, fuel tumor plasticity, metastasis, and therapeutic resistance. Whether the transitions between cell states can be therapeutically targeted remains unknown. Here, using the epithelial-to-mesenchymal transition (EMT) as a model, we show that the transcription reprogramming during a cell-state transition induces genomic instability through R-loops and transcription-replication conflicts and that the cell-state transition cannot occur without the ATR kinase, a key regulator of the replication stress response. ATR inhibition during EMT not only increased transcription- and replication-dependent genomic instability, but also disrupted transcription reprogramming. Unexpectedly, ATR inhibition elevated R-loop–associated DNA damage at the *SNAI1* gene, a key driver of the transcription reprogramming during EMT, triggering ATM- and Polycomb-mediated transcription repression of *SNAI1*. Beyond *SNAI1*, ATR also suppressed R-loops and antagonized repressive chromatin at a subset of EMT genes. Importantly, inhibition of ATR in tumors undergoing EMT reduced tumor growth and metastasis, suggesting that ATR inhibition eliminates cancer cells in transition. Thus, during EMT, ATR not only protects genome integrity but also enables transcription reprogramming, revealing that ATR is a safeguard of cell-state transitions and a target to suppress tumor plasticity.

## Abstract 2

The ATR kinase protects cells undergoing epithelial-to-mesenchymal transition (EMT) against genomic instability and enables transcription reprogramming, making ATR an attractive target to suppress tumor plasticity.

## Introduction

Transitions between cell states are critical for development, tumor plasticity, and evolution (1–3). As the plasticity and evolvability of tumors represent major challenges to cancer therapy today, strategies to suppress these tumor properties are urgently needed. During cell-state transitions, a large number of genes are turned on or off, resetting the transcription programs of cells and their properties and identities. One of the best characterized cell-state transitions is the epithelial-to-mesenchymal transition (EMT) (4–6). During EMT, epithelial cells forego their epithelial characteristics and acquire mesenchymal features with distinct cytoskeletal architecture, allowing the remodeling of cell–cell and cell–extracellular matrix interactions. EMT is not only required for normal embryogenesis, organ development, and wound healing, but also contributes to malignant progression, immunosuppression, metastasis, and therapeutic resistance (4, 7). EMT is driven by transcription reprogramming that involves specific transcription factors, including SNAI1, TWIST, and ZEB1. Notably, cells undergoing EMT display both genomic instability and compromised nuclear structures (8), showing that this cell-state transition, which is associated with rapid and large-scale transcriptional changes, is a stressful process. Here, using EMT as a model, we investigate whether cancer cells undergoing cell-state transitions can be targeted, which could provide a strategy to suppress tumor plasticity and evolution. In particular, we examine whether the genomic instability associated with EMT is a result of transcription reprogramming. Furthermore, we ask how cells respond to genomic instability during EMT and whether this response affects the process of EMT. Finally, we explore whether disrupting the cellular response to EMT-associated genomic instability diminishes cancer metastasis, testing the possibility of impeding tumor progression by targeting cancer cells in transition.

Ataxia telangiectasia mutated (ATM) and ataxia telangiectasia and Rad3-related (ATR) checkpoint kinases are the 2 master regulators of the DNA damage response in human cells (9–11). While ATM primarily responds to DNA double-strand breaks (DSBs), ATR reacts to a broad range of DNA lesions and replication problems that give rise to ssDNA (12). Through their functions in regulating DNA repair, DNA replication, transcription, and the cell cycle, ATM and ATR play critical roles in suppressing genome instability and protecting genome integrity. In cancer cells, genomic instability arises from multiple sources (13–15), including collisions between transcription and DNA replication machineries, known as transcription-replication conflicts (TRCs) (16, 17). TRCs are exacerbated by R-loops, which are typically formed by stable DNA-RNA hybridization during transcription (18–20). In cancer cells driven by the transcription programs established by oncoproteins and oncogenic pathways, such as MYC and the estrogen signaling pathway, R-loop–associated DNA damage is increased (21, 22). Whether the transcription reprogramming during cell-state transitions is sufficient to exert similar effects remains unknown. We have previously shown that ATR is a key sensor and suppressor of R-loop–associated genomic instability (23). Nonetheless, it remains unknown whether ATR or ATM is engaged in an R-loop response during EMT. Furthermore, how the response to genomic instability during EMT affects this cell-state transition is not understood, leaving several possibilities open. For example, it is possible that EMT-associated genomic instability triggers an ATR- or ATM-mediated checkpoint response and blocks the transition. Alternatively, the activation of ATR and/or ATM during EMT may protect cells against genomic instability and allow them to progress through the transition. Understanding the causes of genomic instability during EMT, the roles of ATR and ATM in the response to EMT-associated genomic instability, and the impact of ATR and ATM on the cell-state transition will help address these important questions.

In this study, we found that the genomic instability arising during EMT is dependent on transcription, R-loops, and DNA replication, suggesting that it is caused by TRCs at R-loops during transcription reprogramming. Furthermore, ATR plays a key role in suppressing R-loop–associated genomic instability during EMT. Inhibition of ATR in cells undergoing EMT results in a drastic reduction in the efficiency of EMT, showing that ATR protects cells against genomic instability and enables them to progress through the transition. Unexpectedly, the expression of SNAI1, a key transcription factor driving EMT, is regulated by ATR. In the absence of ATR activity, aberrant R-loops accumulate at the *SNAI1* gene, leading to DNA damage and transcription repression mediated by ATM, Polycomb Repressive Complex 1 (PRC1), and PRC2. Genome-wide analysis of R-loops during EMT revealed that ATR suppresses R-loops at a group of EMT genes, suggesting a broader role for ATR in transcription reprogramming. Using an in vivo tumor model in which EMT can be induced, we found that ATR inhibition during EMT not only reduces the growth of primary tumors, but also diminishes metastasis. Together, our results uncover surprising functions of ATR in transcription reprogramming and safeguarding cells through EMT, suggesting that ATR inhibition may be an effective way to target cancer cells in cell-state transitions and impede tumor plasticity and evolution.

## Results

### EMT is associated with DNA replication stress and DNA damage.

**Figure 1:**
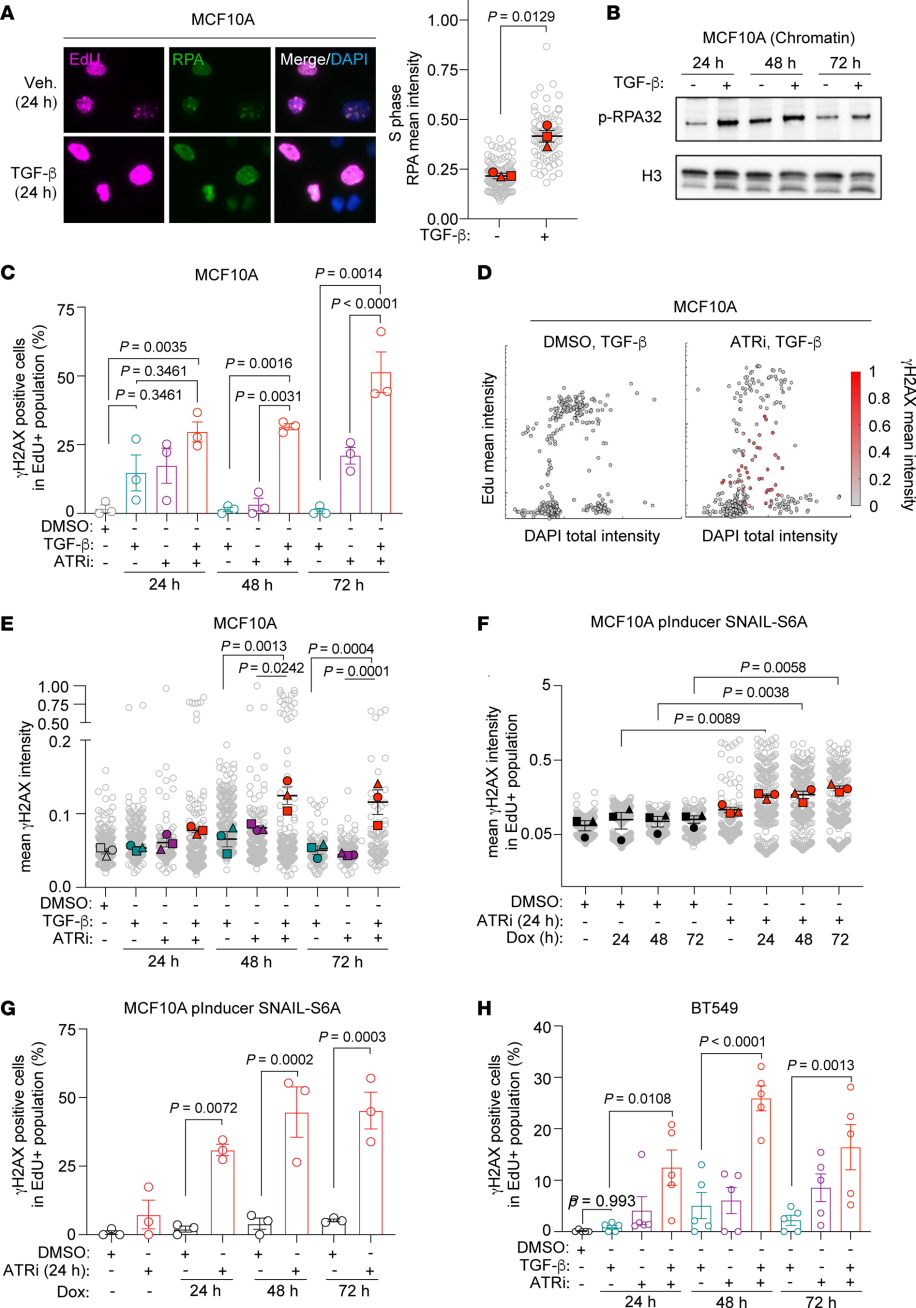
EMT is associated with DNA replication stress and DNA damage. (**A**) Representative immunofluorescence images (left) and quantification of S phase mean RPA32 intensity (right) in MCF10A cells treated with 5 ng/mL TGF-β for 24 h. Original magnification, ×20. (**B**) Western blotting of p-RPA32 (Ser33) and histone H3 in MCF10A cells treated with 5 ng/mL TGF-β for 24, 48, and 72 h. H3 was used as a loading control. (**C**) Quantification of γH2AX^+^ cells in EdU^+^ cell populations from the immunofluorescence experiments performed in MCF10A cells treated with 5 ng/mL TGF-β and/or 10 μM ATRi (VE821) for 24, 48, and 72 h. (**D**) Two-dimensional plot of the cells in **A** displaying total DAPI intensity and mean EdU intensity. Coloring of dots indicates γH2AX mean intensity. (**E**) Quantification of mean γH2AX intensity from the immunofluorescence experiment in **C** and **D**. (**F**) Quantification of nuclear γH2AX intensity in EdU^+^ population from immunofluorescence experiments performed in MCF10A cells expressing pInducer20 SNAIL1-S6A, treated with 50 ng/mL doxycycline to induce SNAIL1-S6A expression for 0, 24, 48, or 72 h and DMSO or 10 μM ATRi (VE821) for the last 24 h. (**G**) Quantification of γH2AX^+^ cells in EdU^+^ cell populations in indicated cells. (**H**) Quantification of γH2AX^+^ cells in EdU^+^ cell population from immunofluorescence experiments performed in BT549 cells treated with 5 ng/mL TGF-β for 0, 24, 48, or 72 h and at the same time DMSO control or 10 μM ATRi (VE821). Statistical significance was determined using a paired 2-tailed *t* test for **A**; 1-way ANOVA for **C**, **G**, and **H**; and 1-way ANOVA followed by Tukey’s multiple-comparison test for **E** and **F**. Data are presented as mean ± SEM in all graphs for 3–5 biological replicates. For scatterplots in **A**, **E**, and **F**, each replicate is indicated by a different symbol. *P* values are indicated in the figure.

To investigate whether the transcription reprogramming during EMT generates replication stress and genomic instability, we used TGF-β to induce EMT in MCF10A breast epithelial cells (24). Replication protein A (RPA) is a heterotrimeric complex of RPA70, RPA32, and RPA14 proteins that specifically binds to ssDNA during DNA replication and repair (25). RPA is not only essential for DNA replication, but also critical for sensing replication stress, activating ATR, and promoting DNA repair. The increase of RPA on chromatin in S phase cells is a common indicator of replication stress (26). Upon a 24 h TGF-β treatment, an increase of chromatin-bound RPA32 was detected by immunofluorescence (Figure 1A). Furthermore, phosphorylated RPA32, which is induced by replication stress, was increased on chromatin (Figure 1B). This marker of replication stress persisted until 72 h after TGF-β exposure (Figure 1B), consistent with the timing of transcription reprogramming during EMT (27). Thus, TGF-β, which activates the transcription program leading to EMT, also increases replication stress during the same time frame.

Next, we asked whether the effects of TGF-β on genome stability are mediated by the SMAD signaling pathway, which is activated by TGF-β and drives EMT. Upon activation by TGF-β, TGF-β receptors phosphorylate SMAD2/3 and enable them to form complexes with SMAD4 (28, 29). The resulting SMAD2-4 and SMAD3-4 complexes then translocate into the nucleus to activate EMT genes such as *SNAI1*, *TWIST*, and *ZEB1*, leading to repression of epithelial genes and activation of mesenchymal genes (30). As expected, after knockdown of SMAD4 by shRNA, SNAI1 was no longer induced by TGF-β in MCF10A cells (Supplemental Figure 1A; supplemental material available online with this article; https://doi.org/10.1172/JCI192225DS1). When nuclear γH2AX intensity was analyzed in individual cells, SMAD4 knockdown modestly increased baseline γH2AX levels after 2 days, but TGF-β did not increase γH2AX levels or the fraction of γH2AX^+^ cells in S phase in the SMAD4-depleted cell populations (Supplemental Figure 1, B and C). Thus, as EMT, TGF-β–induced DNA damage is also mediated by the SMAD pathway, suggesting that DNA damage is associated with EMT.

The induction of replication stress by TGF-β prompted us to test whether ATR plays a role in limiting the EMT-associated DNA damage. Inhibition of ATR during TGF-β treatment with the ATR inhibitor (ATRi) VE821 significantly increased γH2AX levels and the fraction of γH2AX^+^ cells in S phase compared with TGF-β or ATRi treatment alone (Figure 1, C–E), supporting the idea that ATR suppresses DNA damage during EMT. Notably, after treatment with TGF-β and ATRi, the cells with high levels of γH2AX were in S phase but they did not incorporate 5-ethynyl-2′-deoxyuridine (EdU) efficiently (Figure 1D), suggesting that ATRi-induced DNA damage accumulates in cells undergoing EMT and replication simultaneously and that damaged cells failed to continue DNA synthesis efficiently. Importantly, the induction of γH2AX by ATRi was blocked by SMAD4 depletion (Supplemental Figure 1, B and C), confirming that the ATRi-induced DNA damage is also mediated by the SMAD pathway. Based on these results, we posit that ATR suppresses DNA damage formation in cells undergoing both EMT and DNA replication.

To verify our findings beyond TGF-β–induced EMT, we sought to induce EMT independently of TGF-β. Expression of SNAI1 in MCF10A cells directly activates the EMT process (31). Similar to TGF-β treatment, induction of SNAI1 also modestly increased nuclear γH2AX intensity and γH2AX^+^ cells in S phase, and this increase was significantly stimulated by ATRi (Figure 1, F and G). Furthermore, we extended our experiments from MCF10A to the breast cancer cell line BT549. Similar to that in MCF10A cells, TGF-β treatment of BT549 cells increased γH2AX^+^ cells in S phase, and this increase was significantly enhanced by ATRi (Figure 1H). These results confirm that our findings are not limited to TGF-β treatment and MCF10A cells, and they are also applicable to the EMT of cancer cells.

### ATR is required for efficient EMT.

**Figure 2:**
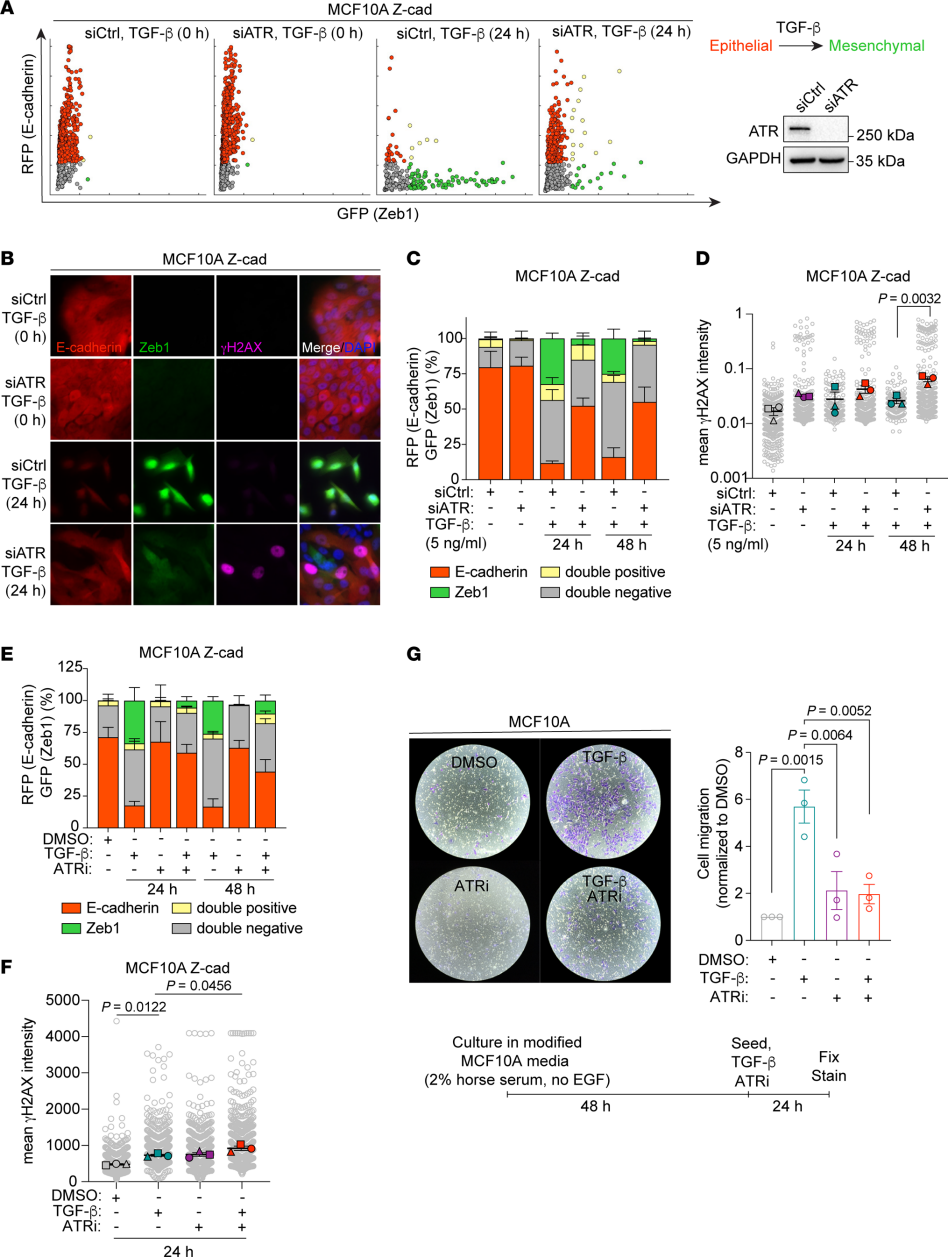
ATR is required for efficient EMT. (**A**) Scatterplot of immunofluorescence experiment in MCF10A Z-cad cells. Cells were treated with siCtrl or siATR-1 for 48 h, then treated with 5 ng/mL TGF-β for an additional 0, 24, or 48 h, then fixed and stained for GFP, RFP, γH2AX, and DAPI. Knockdown efficiency of siATR-1 is shown on the right. (**B**) Representative immunofluorescence images of experiment in **A**. Original magnification, ×20. (**C**) Bar graph of cells from **A** and **B**, color coded for single positive GFP (ZEB1) or RFP (E-cadherin), double positive for both, or double negative for no staining. (**D**) Quantification of mean γH2AX intensity from the immunofluorescence experiment in **A**–**C**. (**E**) Bar graph of MCF10A Z-cad cells treated with 5 ng/mL TGF-β for 0, 24, or 48 h and at the same time DMSO or 1 μM AZD6738, color coded for single positive GFP (ZEB1) or RFP (E-cadherin), double positive for both, or double negative for no staining. Cells were fixed and stained for GFP, RFP, γH2AX, and DAPI. (**F**) Quantification of mean γH2AX intensity from the immunofluorescence experiment at 24 h in **E**. (**G**) Representative images and quantification of transwell migration (normalized to DMSO) in MCF10A cells treated for 24 h with 5 ng/mL TGF-β, 1 μM AZD6738, or both. Statistical significance was determined using 1-way ANOVA followed by Tukey’s multiple-comparison test for **D** and **F** and 1-way ANOVA for **G**. Data are presented as mean ± SEM in all graphs for 3 biological replicates. For scatterplots in **D** and **F**, each replicate is indicated by a different symbol. *P* values are indicated in the figure.

Given the function of ATR in suppressing DNA damage during EMT, we asked whether ATR is important for the process of EMT. Expression of destabilized GFP with the *ZEB1* 3′ UTR and red fluorescent protein (RFP) from the E-cadherin (*CDH1*) promoter established a dual-color reporter of EMT, known as the Z-cadherin (Z-cad) dual sensor (32). Using the Z-cad sensor in MCF10A cells, we tested the impact of ATR loss on EMT. In the absence of TGF-β, the majority of Z-cad cells expressed RFP, reflecting their epithelial state (Figure 2A). Upon TGF-β treatment, the majority of Z-cad cells changed from red to green within 24 h (Figure 2A), showing the efficient occurrence of EMT. Knockdown of ATR by 2 distinct siRNAs did not affect the expression of RFP in the absence of TGF-β, but drastically reduced the conversion of RFP^+^ cells into GFP^+^ cells after TGF-β treatment (Figure 2, A–C, and Supplemental Figure 2A), showing that ATR is required for efficient EMT. Notably, a fraction of ATR-knockdown Z-cad cells became γH2AX^+^ after TGF-β treatment (Figure 2B), suggesting that loss of ATR allows DNA damage to accumulate in cells attempting to undergo EMT. Furthermore, ATR depletion increased nuclear γH2AX intensity in Z-cad cells after TGF-β treatment (Figure 2D). Consistent with the effects of ATR knockdown, treatment of Z-cad cells with 2 ATRis (AZD6378 and VE821) also drastically reduced the conversion of RFP^+^ cells into GFP^+^ cells and increased nuclear γH2AX intensity after TGF-β exposure (Figure 2, E and F, and Supplemental Figure 2B), confirming the ATR specificity of these effects.

Next, we sought to assess the impact of ATR on cell invasion, an EMT-dependent process, functionally. Using the transwell migration assay, we observed that TGF-β treatment increased the transwell migration of MCF10A cells by 5- to 7-fold (Figure 2G). While ATRi alone did not alter cell invasion, it significantly reduced the transwell migration of MCF10A cells after TGF-β treatment (Figure 2G). To corroborate these findings, we performed in vitro scratch assays with BT549 and T47D cells and observed significant reductions in cell invasion in cells undergoing TGF-β–mediated EMT in the presence of ATRi (Supplemental Figure 2C). Taken together, these data highlight an important role of ATR in enabling cells to progress through EMT.

### ATR suppresses DNA damage during EMT by limiting R-loops and TRCs.

**Figure 3:**
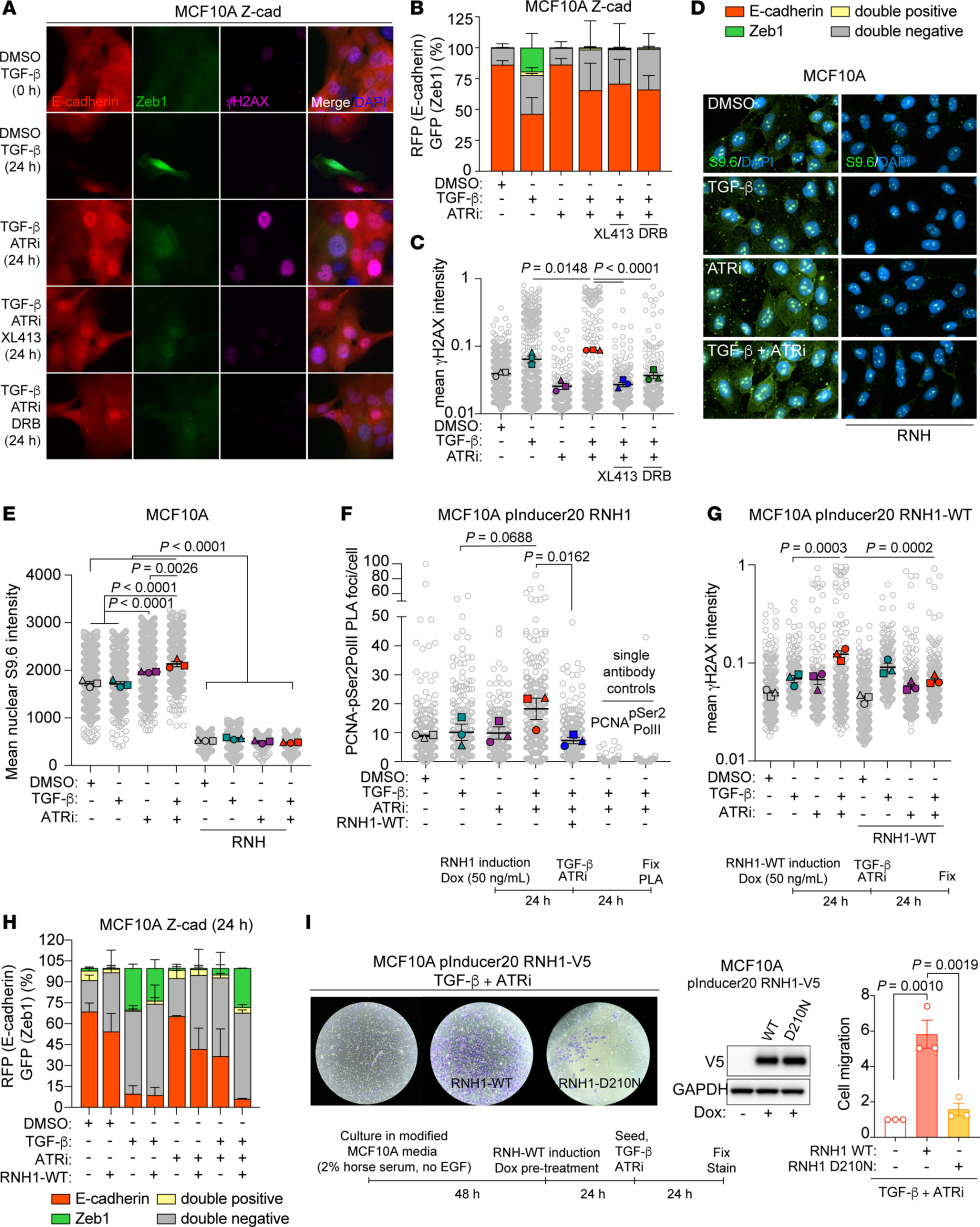
ATR suppresses DNA damage during EMT by limiting R-loops and TRCs. (**A**) Representative images of immunofluorescence in MCF10A Z-cad cells treated with 5 ng/mL TGF-β for 0 or 24 h in combination with either DMSO, 1 μM AZD6738, 5 μM XL413, or 10 μM DRB. Original magnification, ×20. (**B**) Bar graph of cells from the experiment in **A**. (**C**) Quantification of mean γH2AX intensity from **A** and **B**. (**D**) Representative images of S9.6 and DAPI in MCF10A cells treated with DMSO, 5 ng/mL TGF-β, 1 μM AZD6738, or both for 24 h. Original magnification, ×20. (**E**) Quantification of nuclear S9.6 intensity from the experiment in **D**. (**F**) Quantification of PCNA-pSer2 Pol II PLA signal in the indicated cell populations treated with 5 ng/mL TGF-β and/or 1 μM AZD6738 for 24 h. (**G**) Quantification of mean γH2AX intensity in the indicated cell populations treated with 5 ng/mL TGF-β, 1 μM AZD6738, or both for 24 h. (**H**) Bar graph of MCF10A Z-cad control and Z-cad pInducer RNaseH1-WT cells treated with DMSO, 5 ng/mL TGF-β, and/or 1 μM AZD6738. Doxycycline was used to induce RNaseH1-WT for 24 h prior to treatment. (**I**) Representative images, Western blot of RNaseH1 expression, and quantification of transwell migration assay in indicated cells. 50 ng/mL of doxycycline was used to induce RNaseH1 24 h prior to indicated treatment. Statistical significance was determined using 1-way ANOVA followed by Tukey’s multiple-comparison test for **C**, **E**, and **G**; 1-way ANOVA followed by Šidák’s multiple-comparison test between TGF-β and TGF-β+ATRi, and TGF-β+ATRi and TGF-β+ATRi+RNH1-WT for **F**; and 1-way ANOVA for **I**. Data are presented as mean ± SEM in all graphs. For scatterplots, mean of each replicate is indicated by a different symbol. Three biological replicates were performed for each experiment. *P* values are indicated in the figure.

The induction of γH2AX by ATRi in S phase cells undergoing EMT suggests that DNA damage may arise from TRCs. Indeed, in the population of MCF10 Z-cad cells treated with TGF-β and ATRi, few cells displayed GFP (ZEB1) signals, and GFP^+^ and γH2AX^+^ cells were mutually exclusive (Figure 3A), suggesting that ATRi-induced DNA damage is associated with a failure to progress through EMT. Inhibition of transcription by the RNA polymerase II inhibitor 5,6-dichlorobenzimidazole 1-β-d-ribofuranoside (DRB) or suppression of DNA replication origin firing by the CDC7 inhibitor XL413 did not alter the effect of ATRi on TGF-β–induced EMT (Figure 3B), but reduced the ATRi-induced DNA damage in these cells (Figure 3C), suggesting that ATRi induces DNA damage in TGF-β–treated cells in a transcription- and replication-dependent manner. To examine whether R-loops are responsible for the induction of DNA damage by ATRi during EMT, we used the S9.6 antibody to analyze R-loops in cells treated with or without TGF-β and ATRi (Figure 3, D and E, and Supplemental Figure 3A). In additional to quantifying nuclear S9.6 signals, we specifically measured the levels of nucleoplasmic R-loops by subtracting nucleolar S9.6 signals (nucleoli marked by nucleolin) from nuclear S9.6 signals (Supplemental Figure 3A). A modest increase of nuclear R-loops was detected in cells treated with ATRi, and this increase was enlarged in cells treated with both TGF-β and ATRi (Figure 3, D and E, and Supplemental Figure 3A). Importantly, the intensity of nuclear S9.6 signals in all samples was drastically reduced by RNaseH treatment after cell fixation (Figure 3E), confirming the R-loop specificity of S9.6 staining. Thus, during EMT, the ATRi-induced DNA damage is associated with an increase in R-loops.

To directly test whether ATRi induces DNA damage during EMT by increasing R-loop–mediated TRCs, we sought to remove R-loops in cells by inducibly expressing WT RNaseH1. To analyze TRCs, we performed proximity ligation assays (PLAs) with an antibody against proliferating cell nuclear antigen (PCNA), a component of replication fork, and another antibody against the phosphorylated Ser2 of RNAPII, a marker of elongating transcription complex. We posit that collisions between replication and transcription complexes bring PCNA and elongating RNAPII in close proximity, allowing the 2 antibodies to generate PLA signals. In MCF10A cells, PLA foci were detected only when both antibodies were applied, but not when single antibodies were used, confirming the specificity of this assay (Figure 3F). The induction of EMT by TGF-β did not increase PLA foci substantially, but PLA foci were increased by the combination of TGF-β and ATRi (Figure 3F), consistent with the elevation of R-loop levels after ATRi treatment. Importantly, induction of RNaseH1 expression significantly reduced PLA foci (Figure 3F), showing that the TRCs are largely driven by R-loops. Consistent with the idea that R-loops cause TRCs and DNA damage during EMT, expression of RNaseH1 suppressed the ATRi-induced γH2AX in TGF-β–treated cells (Figure 3G). Together, these results establish that ATR suppresses DNA damage during EMT by limiting R-loops and TRCs.

Given the results above, we asked whether R-loop accumulation underlies ATRi-induced failure of EMT. Indeed, induction of RNaseH1 expression in MCF10A Z-cad cells reversed the inhibitory effects of ATRi on EMT (Figure 3H and Supplemental Figure 3, B and C), suggesting that R-loop accumulation interferes with EMT when ATR is inhibited. In addition, in a cell invasion assay, expression of WT RNaseH1 (RNaseH1-WT), but not the catalytically inactive RNaseH1-D210N mutant, significantly rescued the TGF-β–induced transwell migration of MCF10A cells in the presence of ATRi (Figure 3I), showing that the functional impact of ATRi on EMT is also dependent on R-loops. These results lend further support to the notion that ATR functionally facilitates EMT by restricting R-loops and derived DNA damage.

### ATR enables SNAI1 expression during EMT.

**Figure 4:**
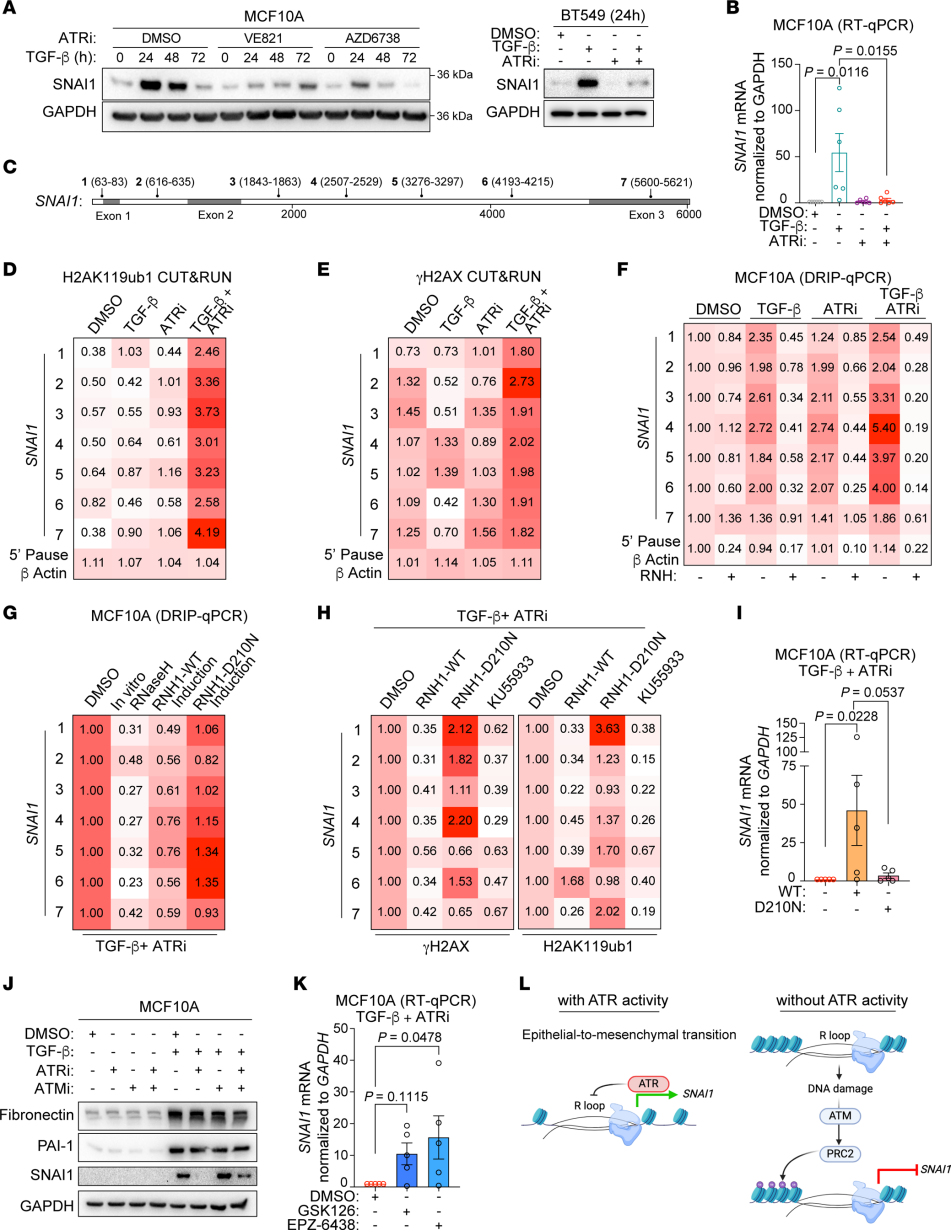
ATR enables *SNAI1* expression during EMT. (**A**) Western blot of SNAI1 in indicated cells treated with TGF-β and various ATRis (10 μM) at indicated time points. (**B**) Bar graph showing *SNAI1* mRNA in the indicated cell populations. (**C**) Schematic of *SNAI1* indicating primer location for CUT&RUN qPCR and DRIP-qPCR. (**D**) Heatmap showing average H2AK119ub1 enrichment via CUT&RUN qPCR at various *SNAI1* loci shown in **C** in MCF10A cells. (**E**) Heatmap of average γH2AX enrichment via CUT&RUN treated and quantified as in **D**. (**F**) Heatmap of average S9.6 enrichment via DRIP-qPCR at various *SNAI1* loci in MCF10A cells. Mean signal normalized to input for each condition and then to DMSO. (**G**) Heatmap showing average S9.6 enrichment via DRIP-qPCR at various *SNAI1* loci in indicated cells. (**H**) Heatmap showing average γH2AX (left) and H2AK119ub1 (right) enrichment via CUT&RUN qPCR at various *SNAI1* loci in the indicated cell populations treated with TGF-β and AZD6738 and/or 10 μM ATMi KU55933. (**I**) Bar graph showing SNAI1 mRNA in the indicated MCF10A cell populations. (**J**) Western blot of the indicated proteins in MCF10A cells treated with TGF-β and AZD6738 and/or 10 μM ATMi KU55933 for 24 h. (**K**) Bar graph showing SNAI1 mRNA in MCF10A cells treated TGF-β and AZD6738 and/or indicated EZH2 inhibitors (10 μM). (**L**) Schematic of proposed model. Statistical significance was determined using 1-way ANOVA for **B**, **I**, and **K**. Three to six biological replicates were performed for each experiment. Doxycycline (50 ng/mL) was used to induce RNaseH1 for 24 h prior to treatment. The 5′ pause region of *ACTB* was used as a control. For experiments in **B**–**K**, cells were treated for 24 h with 5 ng/mL TGF-β and 1 μM AZD6738. *P* values are indicated in the figure.

While the results above suggest that ATR suppresses DNA damage during EMT, why the process of EMT is blocked by ATRi remains unclear. To investigate how ATRi blocks EMT, we analyzed the effects of ATRi on SNAI1, a key transcription factor driving the transcription reprogramming during EMT. In the absence of ATRi, TGF-β treatment induced a robust but transient (24–48 h) accumulation of SNAI1 protein (Figure 4A), consistent with the function of SNAI1 during EMT. In striking contrast, 2 distinct ATRis drastically reduced the induction of SNAI1 protein by TGF-β in MCF10A and BT549 cells (Figure 4A). To examine whether this suppression occurred at the transcription level, we quantified *SNAI1* mRNA using RT-qPCR (Figure 4B). Consistent with the known effects of TGF-β, *SNAI1* mRNA was robustly induced by TGF-β in the absence of ATRi. In the presence of ATRi, however, the induction of *SNAI1* mRNA was abolished (Figure 4B), showing that ATR is required for this key transcription event driving EMT.

The unexpected finding that ATR is required for the transcription activation of *SNAI1* during EMT raised the possibility that the local transcription regulation at the *SNAI1* locus is altered by ATRi. The repressive chromatin mark histone H2A lysine 119 monoubiquitination (H2AK119ub1) is implicated in the transcription repression after DNA damage (33–35). To understand why *SNAI1* expression is not induced by TGF-β in the presence of ATRi, we analyzed H2AK119ub1 at the *SNAI1* locus by cleavage under targets and release using nuclease (CUT&RUN). We performed qPCR with multiple primer pairs across the *SNAI1* locus to follow the changes of H2AK119ub1 levels (Figure 4C). Neither TGF-β nor ATRi alone affected H2AK119ub1 levels (Figure 4D and Supplemental Figure 4A). However, when TGF-β–treated cells were exposed to ATRi, H2AK119ub1 levels were substantially increased at the *SNAI1* locus (Figure 4D and Supplemental Figure 4A), showing that ATRi induces repressive chromatin at the *SNAI1* gene when cells try to activate the transcription program driving EMT. In contrast to the *SNAI1* locus, TGF-β and ATRi did not induce H2AK119ub1 at the constitutively active β-actin (*ACTB*) gene (Figure 4D and Supplemental Figure 4B), suggesting that the induction of repressive chromatin by ATRi is specific to TGF-β–regulated genes.

### ATRi-induced repression of SNAI1 is dependent on R-loops, ATM, and EZH2.

Given the role of ATR in suppressing DNA damage during EMT, we asked whether the ATRi-induced repression of *SNAI1* is associated with DNA damage at this locus. Using γH2AX CUT&RUN coupled with qPCR, we found that the combination of TGF-β and ATRi, but not TGF-β or ATRi alone, induced an increase of DNA damage at the *SNAI1* locus (Figure 4E and Supplemental Figure 4C). In contrast, no increase of γH2AX was observed at the *ACTB* gene after a combined TGF-β and ATRi treatment (Figure 4E and Supplemental Figure 4D). Thus, ATRi indeed induces local DNA damage at the *SNAI1* locus during EMT.

Because ATR suppresses R-loop–associated DNA damage during EMT, next we tested whether R-loops accumulate at the *SNAI1* locus after TGF-β and ATRi treatments. Using DNA-RNA immunoprecipitation (DRIP) coupled with qPCR, we found that either TGF-β or ATRi alone induced a modest increase of R-loops at the *SNAI1* gene (Figure 4F and Supplemental Figure 4E). The levels of R-loops were further increased when TGF-β and ATRi were combined (Figure 4F and Supplemental Figure 4E). Importantly, all DRIP-qPCR signals at the *SNAI1* locus were eliminated by RNaseH treatment, confirming the specificity of this assay (Figure 4, F and G, Supplemental Figure 4E, and Supplemental Figure 5A). Notably, no increase of R-loops was detected at the 5′ pause site of the *ACTB* gene, which is known to harbor constitutive R-loops (Figure 4F), suggesting that the increase of R-loops specifically occurs at TGF-β–regulated genes. These results raise the possibility that TGF-β and ATRi induce R-loop accumulation at the *SNAI1* locus, leading to DNA damage and transcription repression of *SNAI1*. To test this possibility, we induced the expression of RNaseH1-WT or RNaseH1-D210N in the cells treated with TGF-β and ATRi. Expression of RNaseH1-WT reduced the levels of R-loops, γH2AX, and H2AK119ub1 at the *SNAI1* locus (Figure 4, G and H, and Supplemental Figure 5, A–C). In contrast, RNaseH1-D210N, which may trap and stabilize R-loops, increased R-loops, γH2AX, and H2AK119ub1 (Figure 4, G and H, and Supplemental Figure 5, A–C). Importantly, expression of RNaseH1-WT increased *SNAI1* mRNA and SNAI1 protein in the presence of TGF-β and ATRi (Figure 4I and Supplemental Figure 5D), showing that R-loops are indeed the cause of *SNAI1* repression. Together, these results suggest a model in which ATRi induces R-loop–associated DNA damage at the *SNAI1* locus during EMT, causing local transcription repression and a failure of EMT.

Previous studies suggested that DSBs induce local transcription repression in an ATM-dependent manner (33). In this process, ATM promotes the recruitment of PRC1 and PRC2 to DSBs to establish repressive chromatin (34, 35). Consistent with the role of ATM in mediating DNA damage–induced transcription repression, the ATM inhibitor (ATMi) KU55933 reduced the levels of γH2AX and H2AK119ub1 at the *SNAI1* locus in cells treated with TGF-β and ATRi (Figure 4H), and it partially reversed the ATRi-induced reduction of SNAI1 protein in cells undergoing EMT (Figure 4J). In addition, 2 inhibitors of EZH2 (GSK126 and EPZ-6438), a key histone methyl transferase in PRC2, reduced H2AK119ub1 at the *SNAI1* locus and increased *SNAI1* mRNA and SNAI1 protein (Figure 4K and Supplemental Figure 5, D and E). Thus, when ATR is functional, it suppresses R-loop–associated DNA damage at the *SNAI1* locus to enable SNAI1 induction during EMT (Figure 4L). In the absence of ATR activity, the initial transcription activation of *SNAI1* leads to R-loop accumulation and local DNA damage, which triggers ATM- and PRC1/2-mediated transcription repression at this locus, contributing to the failure to progress through EMT (Figure 4L).

### ATR suppresses R-loops at a group of EMT genes.

**Figure 5:**
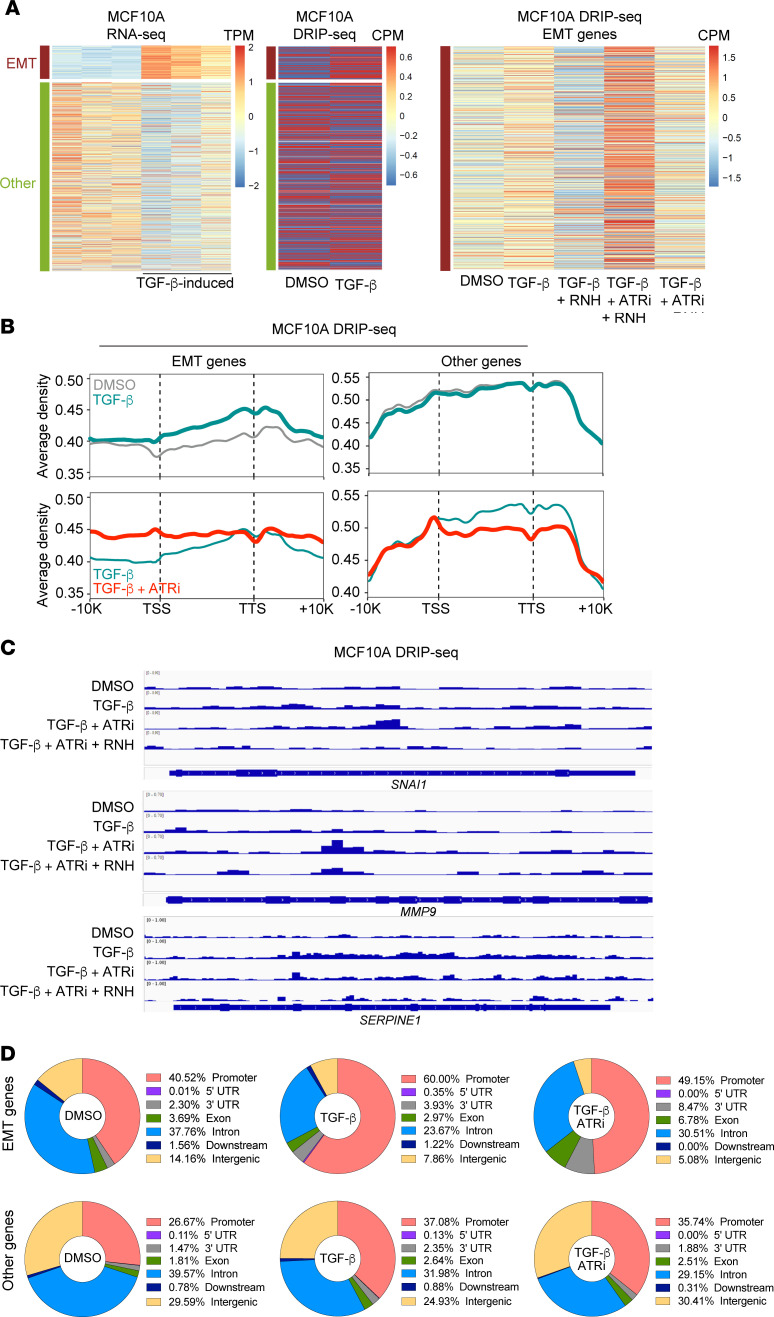
ATR suppresses R-loops at a group of EMT genes. (**A**) Heatmap showing TGF-β–induced transcripts from RNA-seq data grouped by EMT genes and other (non-EMT) genes (left) after 24 h. Heatmap of DRIP-seq data at genes shown in panel on the left in MCF10A cells 24 h after TGF-β treatment (middle). Heatmap of DRIP-seq data at EMT genes (right). For DRIP-seq, MCF10A cells were treated with DMSO, 5 ng/mL TGF-β, 1 μM AZD6738, or both for 24 h. TPM, transcripts per million; CPM, counts per million. (**B**) Metagene plots of average R-loop density in MCF10A cells treated with DMSO, 5 ng/mL TGF-β, 1 μM AZD6738, or both for 24 h. EMT genes refer to genes induced by TGF-β from RNA-seq data in **A**. TSS, transcription start site; TTS, transcription termination site. (**C**) Gene tracks of EMT genes *SNAI1*, *MMP9*, and *SERPINE1* in indicated DRIP-seq samples. (**D**) Distribution of peaks from DRIP-seq in MCF10A cells treated with DMSO, 5 ng/mL TGF-β, 1 μM AZD6738, or both for 24 h.

To investigate the impact of ATR on R-loops in the genome during EMT more comprehensively, we performed DRIP-sequencing (DRIP-seq) in MCF10A cells treated with or without TGF-β and ATRi for 24 h. At a group of genes activated by TGF-β during EMT (27), R-loop levels were increased after TGF-β treatment (Figure 5, A and B). In contrast, no increase of R-loops was observed at non-EMT genes expressed at similar levels (Figure 5B). At the EMT genes, R-loop levels were further increased by the combined treatment with TGF-β and ATRi compared with TGF-β treatment alone, whereas no such increase was detected at non-EMT genes (Figure 5, A and B). The ATRi-induced increase of R-loops at EMT genes was observed throughout gene bodies and flanking intergenic regions. Examination of DRIP-seq signals at specific loci confirmed the ATRi-induced increase of R-loops at EMT genes such as *SNAI1* and *MMP9* (Figure 5C). However, at some other EMT genes, such as *SERPINE1*, ATRi did not increase the overall levels of R-loops (Figure 5C). R-loops at *ACTB*, a non-EMT gene, were not increased by ATRi (Supplemental Figure 6A). Importantly, the DRIP-seq signals at EMT genes and *ACTB* were reduced by RNaseH treatment (Figure 5A and Supplemental Figure 6, A–C), confirming the R-loop specificity of these signals. These results show that ATR not only suppresses R-loops at the *SNAI1* locus, but also at a subset of EMT genes.

To further understand how ATR suppresses R-loops at EMT genes, we analyzed the distribution of R-loop peaks in various parts of gene bodies and intergenic regions. At the EMT genes activated by TGF-β, the fraction of R-loop peaks at promoters was substantially increased after TGF-β treatment (Figure 5D), suggesting an association of promoter R-loops with transcription activation. This increase of promoter R-loops after TGF-β treatment was much less pronounced at non-EMT genes (Figure 5D). Furthermore, in cells treated with both TGF-β and ATRi, the fractions of R-loop peaks in exons, introns, and 3′ UTRs were increased compared with those in cells treated with TGF-β alone (Figure 5D). In contrast, this redistribution of R-loop peaks was not observed at non-EMT genes (Figure 5D). Thus, ATRi specifically alters not only the levels, but also the distribution of R-loops at EMT genes after TGF-β treatment, possibly because ATR is required for removing transcription-coupled or TRC-induced R-loops when cells are trying to activate these EMT genes (36–38).

We also performed H2AK119ub1 CUT&TAG to test how ATRi affects repressive chromatin at EMT genes. As expected, the transcription activation of EMT genes by TGF-β reduced H2AK119ub1 at these genes (Supplemental Figure 6D). After cells were cotreated with TGF-β and ATRi, H2AK119ub1 was increased at EMT genes (Supplemental Figure 6E), consistent with the failure of these cells to progress through EMT. Analysis of H2AK119ub1 at *SNAI1* and *MMP9* confirmed the induction of this repressive histone mark by ATRi in the presence of TGF-β (Supplemental Figure 6F). Interestingly, ATRi also increased H2AK119ub1 at *SERPINE1* (Supplemental Figure 6F), where ATRi did not increase R-loops (Figure 5C). Thus, even at EMT genes where ATR does not suppress R-loop levels, ATR still protects these genes from TGF-β–induced R-loops and antagonizes transcription repression.

### ATR inhibition during EMT in vivo impedes tumor growth and metastasis.

**Figure 6:**
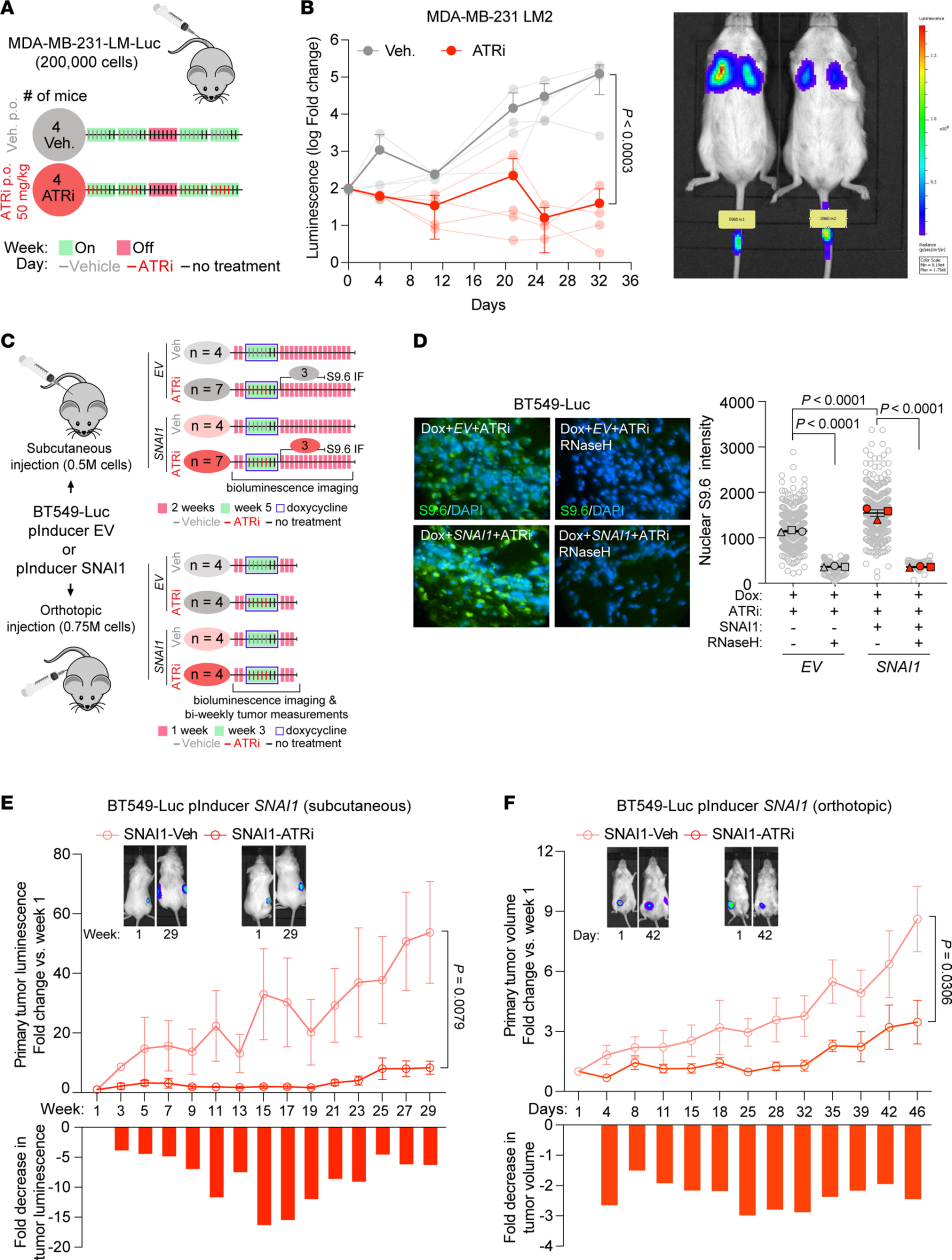
ATR inhibition during EMT in vivo impedes tumor growth and metastasis. (**A**) Schematic of in vivo experiment in **B**. (**B**) Line graph plotting tumor bioluminescence in indicated treatment groups from **A**. Log fold change in tumor bioluminescence was normalized to day 0 (day of tail vein injection). Data are presented as mean ± SEM for 4 mice in each treatment group. Representative images are shown on the right. (**C**) Schematic of the in vivo experiments in **D**–**G**. (**D**) Immunofluorescence of nuclear S9.6 intensity in BT549 subcutaneous tumors. Slides were incubated overnight with RNaseH as control and then subject to immunofluorescence with the S9.6 antibody. Representative images are shown on the left. Quantification of nuclear S9.6 intensity is shown on the right. Original magnification, ×10. (**E**) Line graph plotting primary tumor bioluminescence in the indicated cohorts from the experiment in **C**. Each value represents fold change in respective cohorts compared with measurements from week 1. Data are presented as mean ± SEM for 3–4 mice in each group. The difference in fold change at each time point is plotted below. (**F**) Line graph plotting primary tumor volume in indicated cohorts from the orthotopic injection experiment in **C**. Each value represents fold change in respective cohorts compared with measurements from day 1 of treatment. Data are presented as mean ± SEM for 3–4 mice in each group. The difference in fold change at each time point is plotted below. Statistical significance was determined using repeated measures 2-way ANOVA with Geisser-Greenhouse correction for **B**, **E**, and **F** and 1-way ANOVA followed by Tukey’s multiple-comparison test for **D**, and each replicate is indicated by a different symbol. *P* values are indicated in the figure.

EMT is implicated in multiple steps of metastasis (39). Given that ATRi impaired TGF-β– and SNAI1-induced EMT in vitro, we sought to examine how ATRi impacts metastasis in vivo. First, we tested the effects of ATRi using a well-established lung metastasis assay of breast cancer cells (40). We injected lung-homed MDA-MB-231-LM cells expressing luciferase into the tail vein of NOD-scid IL2Rgamma^null^ (NSG) mice. This cell line is a lung-selective metastatic derivative of the MDA-MB-231 cell line, which was derived from a patient with triple-negative breast adenocarcinoma, and it was genetically engineered to express luciferase, enabling visualization of lung metastasis in vivo (40). Mice were then randomly sorted into 2 cohorts and administered DMSO or 50 mg/kg ATRi (AZD6738) via oral gavage for 5 days per week, for 5 weeks, and with week 3 off (Figure 6A). Through quantification of bioluminescence, we followed the growth of lung metastases over 36 days. We found that mice treated with ATRi had a significant reduction in lung metastatic burden compared with those treated with DMSO (Figure 6B). It should be noted that MDA-MB-231 cells display mesenchymal features (41), and a previous study showed that MDA-MB-231 cells undergo mesenchymal-to-epithelial transition (MET) rather than EMT when they metastasize from primary tumors to the lung (42, 43). Eribulin is known to induce MET in MDA-MB-231 cells (44). Similar to EMT, eribulin-induced MET was also associated with increased γH2AX, which was exacerbated by ATRi (Supplemental Figure 7A). These results suggest that ATRi impedes metastasis in vivo, possibly by reducing tumor cell viability and/or preventing them from going through cell-state transitions.

To assess the effects of ATRi on tumors undergoing EMT directly, we generated a tumor model in which we could conditionally induce EMT. We used BT549 cells expressing luciferase and harboring doxycycline-inducible empty vector (EV) or *SNAI1* and injected the cells into the flanks of NSG mice. Eleven mice were subcutaneously injected with BT549-Luc-pInducer EV cells, and 11 mice were injected with BT549-Luc-pInducer *SNAI1* cells (Figure 6C). Four weeks after injections, all mice were administered doxycycline in drinking water containing sucrose for 1 week. Four mice in each group were administered DMSO, and 7 mice in each group were administered 50 mg/kg ATRi for 5 consecutive days alongside doxycycline. To examine whether SNAI1 induction increases R-loops in tumors, tumors were harvested from 3 mice in each group that received ATRi, fixed, and analyzed by S9.6 staining. We found that R-loop levels in tumors were increased by SNAI1 induction in the presence of ATRi (Figure 6D). Importantly, the S9.6 signals detected in tumors were eliminated by RNaseH treatment (Figure 6D), confirming the specificity of this assay. These results confirm that tumors undergoing EMT in the presence of ATRi indeed accumulate more R-loops.

To determine the effects of ATRi on tumor progression, the remaining mice were monitored biweekly through quantification of tumor bioluminescence to (a) examine the effects of *SNAI1* expression on tumor growth and metastasis, (b) examine whether ATRi affects the growth of primary tumors with or without *SNAI1* induction, and (c) examine whether ATRi affects metastasis. Induction of *SNAI1* expression in tumors reduced the growth of primary tumors but increased metastasis (Table 1, see Supplemental Figure 7C), consistent with induction of DNA damage by EMT and the role of EMT in promoting metastasis. When primary tumors induced to express SNAI1 were compared after treatments with vehicle or ATRi, ATRi substantially reduced tumor growth (Figure 6E), suggesting that tumor cells undergoing EMT in vivo are sensitive to ATRi. Furthermore, when tumors with and without SNAI1 induction were compared after ATRi treatment, the tumors overexpressing SNAI1 grew much more slowly (Supplemental Figure 7B). Importantly, the inhibitory effects of ATRi were more prominent in tumors with SNAI1 induction than those without (Supplemental Figure 7C), suggesting that ATRi preferentially kills tumor cells undergoing EMT in vivo. Thus, even primary tumors are more sensitive to ATRi when they undergo EMT, suggesting that ATRi may suppress EMT-driven tumor plasticity and heterogeneity in primary tumors without the complete process of metastasis.

To test the effects of ATRi on metastasis, we examined the mice for metastases outside of primary tumors with bioluminescence. In the cohort of mice bearing tumors induced to express SNAI1 and treated with vehicle, we found 2 metastases in 4 mice (Table 1). In contrast, no metastases were found in the mice bearing tumors induced to express SNAI1 and treated with ATRi (0 metastasis in 4 mice).

To confirm and extend the above results, we repeated this in vivo experiment using BT549 cells in an NCG orthotopic tumor model (Figure 6C). Consistently, the growth of primary tumors in this orthotopic model was more extensively inhibited by ATRi after SNAI1 induction (Figure 6F and Supplemental Figure 7, D and E), supporting the idea that ATRi preferentially kills tumor cells undergoing EMT in primary tumors. In addition, we observed spontaneous lung metastases even without SNAI1 induction in 3 of 4 mice and both lung and spleen metastases after SNAI1 induction in 3 of 3 mice (Table 1, Figure 6F, and Supplemental Figure 7F). Importantly, spontaneous metastasis was only found in 1 of 4 mice after ATRi treatment, and metastasis from SNAI1-expressing tumors was detected in 1 of 4 mice treated with ATRi (Table 1, Figure 6F, and Supplemental Figure 7F), confirming that ATRi indeed suppresses metastasis in this orthotopic model. These results show that ATRi inhibits EMT-driven metastasis, suggesting that ATR inhibition may be an effective way to target tumor cells undergoing transitions in vivo and impede tumor progression.

## Discussion

The plasticity of cancer cells contributes to tumor heterogeneity, tumor evolution, and therapeutic resistance, presenting a major obstacle to cancer therapy today (1). Cancer cells acquire plasticity through transcription changes, often involving transcription reprogramming of many genes (45). Although transcription reprogramming increases the ability of cancer cells to evolve under selective pressure and obtain malignant properties, it also gives rise to genomic instability (46). Recent studies suggested that transcription activation by oncoproteins or oncogenic pathways is often associated with increases in R-loops and TRCs, resulting in elevated levels of DNA damage. For example, both MYC and HRAS^V12^ oncoproteins were shown to increase R-loops and TRCs (47, 48). Furthermore, estrogen activates transcription of a large group of genes in estrogen receptor^+^ cancer cells, leading to R-loop–associated DNA damage (22). Thus, the oncogenic transcription programs in cancer cells are likely sources of genomic instability and DNA damage. However, whether the transcription reprogramming in cancer cells undergoing cell-state transitions, the transient process driving cancer plasticity, is sufficient to induce substantial genomic instability remains unknown. In addition, it is unclear how cell-state transitions themselves are affected by R-loops and TRCs. In this study, we used EMT as a model to investigate the impact of transcription reprogramming on genome stability and the effects of R-loops and TRCs on cell-state transitions. Our findings reveal a surprising role for ATR in coordinating transcription reprogramming and R-loop regulation during EMT, suggesting that ATR is a key safeguard of cell-state transitions.

Our results show that ATR protects cells undergoing EMT against R-loop–mediated TRCs and DNA damage. In cells induced to undergo EMT, DNA replication stress and DNA damage start to rise during the early phase of transcription reprogramming, and DNA damage is substantially exacerbated by ATRi. The ATRi-induced DNA damage during EMT occurs in S phase and is dependent on both transcription and replication, showing that it is driven by TRCs. Furthermore, ATRi-induced DNA damage is suppressed by RNaseH1, tracing its origin to R-loops. These results are consistent with previous reports that transcription activation increases R-loops, TRCs, and DNA damage (22, 48) and that ATR counters R-loops and TRCs (23, 37, 38). More importantly, they argue that EMT, a cell-state transition driven by transcription reprogramming, is sufficient to generate substantial R-loops and TRCs if not restricted by ATR. How ATR suppresses R-loop–derived genomic instability during EMT is still not fully understood. We observed an increase of R-loops and TRCs after ATRi treatment, suggesting that ATR suppresses R-loop accumulation during transcription reprogramming. Our preliminary analysis suggests that the levels of R-loops at EMT genes are associated with the extents of transcription activation by TGF-β and the GC content of these genes (data not shown). In cells treated with topoisomerase I inhibitor, head-on (HO) collisions between R-loops and replication forks at active genes lead to local accumulation of phosphorylated RPA, a substrate of ATR, suggesting that ATR is activated at these sites (49). In addition, on episomes where HO collisions occur between replication forks and R-loops, R-loop levels are elevated and ATR is activated (50). These results suggest that ATR is critical for suppressing R-loop accumulation at sites of HO collisions. It is plausible that the transcription activation during EMT increases HO collisions at a group of EMT genes, including *SNAI1*, creating a dependency on ATR for suppressing R-loops and TRCs at these sites. In the absence of ATR activity, HO collisions generate more R-loops and DNA damage, making ATR an indispensable safeguard of the transition.

Our results also reveal a surprising role of ATR in controlling the transcription reprogramming during EMT. The transcription activation of *SNAI1* is a key event to initiate EMT. In the presence of functional ATR, *SNAI1* expression is efficiently induced by TGF-β, which is accompanied by a slight increase of R-loops but no increase of DNA damage at this locus. In the absence of ATR activity, however, both R-loops and DNA damage are increased at the *SNAI1* locus, and *SNAI1* expression is not efficiently induced. These results suggest that ATR senses the initial TRCs at the *SNAI1* locus and prevents further accumulation of R-loops and substantial DNA damage at this site, which is necessary for the efficient induction of *SNAI1* expression. Consistent with the model in which ATM promotes Polycomb-mediated transcription repression at DNA damage sites (33–35), the inhibitory effects of ATRi on *SNAI1* expression are dependent on ATM and PRC1/2 complexes. Both H2AK119ub1, a repressive histone mark generated by PRC1, and EZH2, the histone methyltransferase in PRC2, are involved in *SNAI1* repression. Importantly, the expression of *SNAI1* was not only rescued by inhibition of ATM or EHZ2, but also by RNaseH1 expression, suggesting that the ATM- and Polycomb-mediated repression is triggered by R-loops. Thus, by suppressing R-loops and DNA damage at the *SNAI1* locus, ATR counters the local transcription repression by ATM and Polycomb, ensuring the induction of *SNAI1* during EMT. In addition to *SNAI1*, ATR may enable the induction of other EMT genes, especially those facing HO collisions with replication forks, through a similar mechanism. The role of ATR in ensuring the induction of *SNAI1* and similar EMT genes during transcription reprogramming is likely required for the subsequent phases of EMT, making ATR a key regulator of the entire process. These findings raise the possibility that ATR is not just a safeguard of genome stability during cell-state transitions, but also an integral regulator of the transcription reprogramming driving these transitions.

It should be noted that the transcription reprogramming during EMT involves not only activation of mesenchymal genes, but also repression of epithelial genes. SNAI1-mediated transcription repression is associated with an increase of H3K27me3, suggesting the involvement of heterochromatin (31). Recent studies showed that the heterochromatin induced by *IDH1/2* oncogenic mutants and the KRAS^G12V^ oncoprotein increases replication stress and the ATR dependency of cancer cells (51, 52). It is possible that the transcription repression during EMT also alters the epigenetic landscapes of chromosomes and contributes to replication stress. One also needs to keep in mind that EMT gives rise to a variety of intermediate cell states between the epithelial and the mesenchymal state (4), and the transcription changes between these intermediate states may generate variable levels of R-loops and TRCs. Thus, the effects of ATR inhibition may vary in different windows of EMT and in different subpopulations of tumor cells undergoing EMT. Despite the heterogeneity in tumor cells undergoing EMT, ATR inhibition may preferentially eliminate the tumor cells undergoing robust reprogramming, thereby reducing cell-state transitions in tumors and in the process of metastasis.

It is also important to note that ATM and ATR play opposite roles during EMT. Whereas ATR protects cells through EMT, the activation of ATM by DNA damage during EMT triggers transcription repression at genes driving the transition. The role of ATM during EMT is consistent with an R-loop/TRC checkpoint, which blocks the transition in response to genomic instability. However, in contrast to ATM, ATR primarily acts to suppress DNA damage and enable transcription reprogramming, highlighting the distinct roles of ATM and ATR during cell-state transitions.

Given the importance of ATR in EMT, inhibition of ATR may be an effective way to impede EMT of cancer cells and prevent metastasis. Metastasis is a complex, multistep process involving dissemination of cancer cells from primary tumors, penetration of cancer cells into and out of blood vessels, and colonization of cancer cells at distal sites (53, 54). EMT is shown to promote multiple steps during the process of metastasis (55). Indeed, our data show that ATRi significantly reduces EMT-mediated cell invasion in vitro. Furthermore, when cancer cells are induced to undergo EMT in tumors, ATRi reduces tumor growth more efficiently, suggesting that cancer cells undergoing EMT in primary tumors are more susceptible to ATRi. Finally, induction of EMT in primary tumors indeed increases metastasis, and this increase is abolished by ATRi. Collectively, these results suggest that ATRi can be used to impede the process of metastasis. Of note, our results do not exclude the possibility that ATRi can kill cancer cells independently of EMT and metastasis, but the preferential killing of cancer cells undergoing EMT by ATRi argues that ATRi directly eliminates cancer cells during the process of metastasis. Our in vitro results suggest that the effects of ATRi on metastatic cells are dependent on R-loops, providing a model to test in vivo in the future. Interestingly, ATRi reduces metastasis even in a model using MDA-MB-231 cells, which display mesenchymal features. MET, which is required for the colonization of mesenchymal cancer cells at distal sites, is also associated with DNA damage, and cells undergoing MET are also protected by ATR. Besides its functions in protecting the genome against R-loops and TRCs and enabling transcription reprogramming, ATR is also implicated in the response to the mechanical stress during interstitial migration, which is associated with metastasis (56, 57). In addition to metastasis, it is plausible that ATRi can reduce EMT-driven tumor plasticity, tumor evolution, immunosuppression, and therapeutic resistance. The ability of ATRi to impede EMT and eliminate cancer cells in transition may provide a promising strategy to overcome the EMT-related challenges in cancer therapy.

Finally, the function of ATR in cell-state transitions may go beyond EMT and MET. ATR deletion in mouse results in embryonic lethality due to DNA damage in blastocysts (58), which undergo both rapid proliferation and transcription reprogramming. ATR maintains progenitor cells during nervous system development (59), which may be attributed to the transcription reprogramming in these cells. Furthermore, ATR enables embryonic stem cells to differentiate in multiple fates (60), presumably by countering TRCs and replication stress. In tumors, cancer cells often undergo partial dedifferentiation and acquire progenitor or stem cell features, allowing them to change their cell identities and evolve under selective pressure (45). The cell-state transitions of cancer cells in tumors may be key drivers of tumor heterogeneity, plasticity, and evolution, contributing to tumor progression, metastasis, and therapeutic resistance. It is tempting to speculate that the transcription reprogramming during these cell-state transitions is broadly dependent on the roles of ATR in safeguarding the genome and countering R-loop–induced transcription repression. In future studies, it will be important to examine the roles of ATR in various contexts of oncogenic cell-state transitions and test the ability of ATRi to impede metastasis and therapeutic resistance in cancer patients.

## Methods

Additional methods can be found in the supplemental materials.

### Sex as a biological variable.

All cell lines used in this study were derived from nontransformed or cancerous breast epithelium; thus, all mice used in this study were biologically female. Although this study primarily focused on breast cancer, basic mechanistic findings from this study may be extrapolated to other cancer types.

### Statistics.

All statistical analyses were performed using Prism 10 version 10.1.1 (GraphPad Software). The number of biological replicates and statistical tests performed are reported in the figure legends. *P* values are indicated in the figures.

### Study approval.

All experimental procedures using mice were reviewed and approved by the Massachusetts General Hospital Center for Comparative Medicine and performed in compliance with Institutional Animal Care and Use Committee policies.

### Data availability.

All data values supporting the findings of this manuscript are provided in the Supporting Data Values file. All materials generated in this study are available upon request from the corresponding author. Uncropped blots and gels are available in the supplemental materials. This paper does not report any original code.

## Author contributions

PSP, JPM, and LZ designed the study with input from other authors. PSP and JPM performed most experiments and data analysis. XR performed bioinformatic analyses. MS performed some of the mouse experiments. ASK performed the Western blots in Figure 1. MW and JLH prepared libraries for sequencing. SS performed immunofluorescence experiments. EW, CS, and JC, helped with mouse experiments. LZ, SM, NJD, DAH, and RBC supervised the study. PSP and LZ wrote the manuscript with input from other authors. All authors agreed to list PSP as the first of the 2 co–first authors for her contributions to the key experiments and manuscript preparation.

## Conflict of interest

LZ is a scientific advisor for Sirrona Therapeutics and received research support from Calico, Pfizer, and Bristol Myers Squibb.

## Funding support

This work is the result of NIH funding, in whole or in part, and is subject to the NIH Public Access Policy. Through acceptance of this federal funding, the NIH has been given a right to make the work publicly available in PubMed Central.

- Canadian Institutes of Health Research Banting Postdoctoral Fellowship FRN 188253 (to PSP).
- NIH training grant 5T32CA009216-39 (to JPM).
- James A. Harting Scientific Scholar Award from the Rivkin Center (to SS).
- James and Patricia Poitras Endowed Chair in Cancer Research at Massachusetts General Hospital (LZ).
- NIH grants CA248526 and CA263934 (to LZ) and CA220323 to NJD.

## Supplementary Material

Supplemental data

Unedited blot and gel images

Supporting data values

## Figures and Tables

**Table 1:**
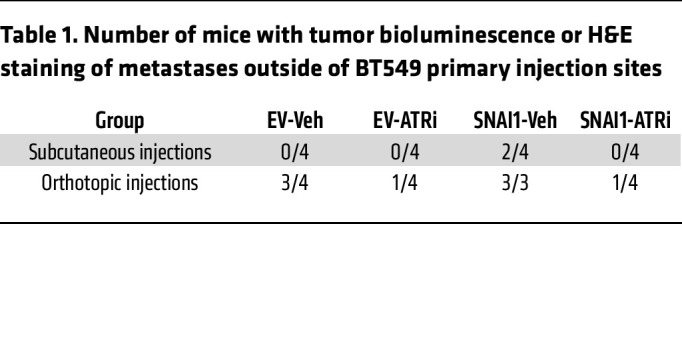
Number of mice with tumor bioluminescence or H&E staining of metastases outside of BT549 primary injection sites

## Version 1 01/22/2026

In-Press Preview

## Version 2 08/17/2026

Electronic publication
