# Supplementary material for: ATR safeguards epithelial-to-mesenchymal transition by countering R-loops and enabling transcription reprogramming
Source: J Clin Invest. 2026 Jan 22;136(16):e192225. doi: 10.1172/JCI192225 (PMC13476143; doi:10.1172/JCI192225)
Supplement: Supplementary file 1 [file jci-136-192225-s121.pdf]

Supplemental Information

Supplemental Figure Legends

Supplemental Figure 1. TGF-β-mediated EMT is dependent on SMAD4

A) Western blot showing SMAD4 and SNAI1 levels in MCF10A control and SMAD4-depleted cells treated with 5 ng/mL TGF-β for 0, 24, 48, and 72 h. GAPDH was used as a loading control.

B) Scatterplot of mean γH2AX intensity from immunofluorescence experiment in control and SMAD4-depleted MCF10A cells treated with 5 ng/mL TGF-β and/or 1 μM AZD6738 for 24 h (left) or 48 h (middle). Cells were pre-extracted with CSK + 0.5% Triton X-100 then fixed and stained for γH2AX and DAPI.

C) Bar graph of S phase (EdU-positive) γH2AX-positive cells from immunofluorescence experiment in control and SMAD4-depleted MCF10A cells treated with 5 ng/mL TGF-β and/or 1 μM AZD6738 for 24 h (left) or 48 h (right). Cells were pre-extracted with CSK + 0.5% Triton X-100 then fixed and stained for EdU, γH2AX and DAPI. γH2AX was quantified in EdU-positive cells.

Statistical analysis was done as in Figure 1.

Supplemental Figure 2. ATR promotes EMT

A) Bar graph of MCF10A Z-cad cells transduced with siCtrl or siATR-2 and treated with 5 ng/mL TGF-β for 24 h, color coded for single positive GFP (ZEB1) or RFP (E-cadherin), double positive for both or double negative for no staining. Cells were fixed and stained for GFP, RFP, γH2AX and DAPI. Mean with standard error shown for three biological replicates.

B) Bar graph of MCF10A Z-cad cells treated with 5 ng/mL TGF-β for 24 h and at the same time DMSO or 10 μM VE821, color coded for single positive GFP (ZEB1) or RFP (E-cadherin), double positive for both or double negative for no staining. Cells were fixed and stained for GFP, RFP, γH2AX and DAPI. Mean with standard error shown for three biological replicates.

C) Bar graphs and representative images of cell invasion at 24 h compared to baseline in BT549 and T47D cells. Cells were scratched using a p200 tip, washed with PBS, and treated with DMSO, 5ng/mL TGF-β, and/or 1 μM AZD6738. Mean with standard error is shown for 3 biological replicates.

Statistical significance for C was determined using a 2-way ANOVA test.

Supplemental Figure 3. ATR promotes EMT by suppressing R loops.

A) Quantification of nucleoplasmic S9.6 immunofluorescence in MCF10A cells treated with 5 ng/mL TGF-β, 1 μM AZD6738, or both, for 24 h. Cells were pre-extracted with 0.5% NP-40 for 3-4 mins and fixed. Coverslips were incubated with RNaseIII and RNaseT1 and stained for S9.6 and nucleolin. Nucleoplasmic S9.6 intensity was quantified by subtracting S9.6 signal in nucleolin-positive regions of the nucleus. Mean with SEM is shown for each replicate.

B) Bar graph of MCF10A Z-cad control and pInducer RNaseH1-WT cells treated with 5 ng/mL TGF-β for 48 (left) or 72 (right) h and at the same time DMSO or 1 μM AZD6738, color coded for single positive GFP (ZEB1) or RFP (E-cadherin), double positive for both or double negative for no staining. Doxycycline was used to induce RNaseH1-WT for 24 h prior treatment with TGF-β and AZD6738 in indicated samples. Cells were

fixed and stained for GFP, RFP, γH2AX and DAPI. Mean with standard error shown for three biological replicates.

C) Scatterplot of mean RNaseH1-WT intensity in MCF10A cells from B and figure 3H.

Statistical analysis was done as in Figure 3.

Supplemental Figure 4. ATRi-induced transcriptional repression of *SNAI1* is dependent on R-loops

A) Violin plots showing average H2AK119ub1 enrichment via CUT&RUN quantified using qPCR at *SNAI1* in MCF10A cells treated for 24 h with 5 ng/mL TGF-β, 1 μM AZD6738, or both. Mean signal with range is shown for 5 biological replicates.

B) Scatter plot showing average H2AK119ub1 enrichment via CUT&RUN quantified using qPCR at the 5' Pause region of *ACTB* in MCF10A cells from experiment in A. Mean with standard error is shown for 5 biological replicates.

C) Violin plots showing average γH2AX enrichment via CUT&RUN quantified using qPCR at *SNAI1* in MCF10A cells treated for 24 h with 5 ng/mL TGF-β, 1 μM AZD6738, or both. Mean signal with range is shown for 5-6 biological replicates.

D) Scatter plot showing average γH2AX enrichment via CUT&RUN quantified using qPCR at the 5' Pause region of *ACTB* in MCF10A cells from experiment in C. Mean with standard error is shown for 5-6 biological replicates.

E) Violin plots showing average S9.6 enrichment via DRIP quantified using qPCR at *SNAI1* in MCF10A cells treated for 24 h with 5 ng/mL TGF-β, 1 μM AZD6738, or both. 5' Pause region of the *ACTB* gene was used as a negative control (data not shown).

Mean signal normalized to input for each condition and then to DMSO for 5 biological replicates is shown. RNaseH treatment controls are also shown.

Statistical analysis was done as in Figure 4.

Supplemental Figure 5. ATRi-induced repression of *SNAI1* is dependent on R-loops, ATM, and EZH2

A) Violin plots showing average S9.6 enrichment via DRIP-qPCR at *SNAI1* in the indicated cells treated for 24 h with 5 ng/mL TGF-β and 1 μM AZD6738. Mean normalized to input for each condition and then to TGF-β+AZD6738 treated sample for 5 biological replicates is shown.

B) Violin plots showing average γH2AX enrichment via CUT&RUN-qPCR at *SNAI1* in the indicated cells treated for 24 h with 5 ng/mL TGF-β and 1 μM AZD6738. ATMi KU55933 (10 μM) was added at the same time as TGF-β and AZD6738. Signal is normalized to IgG and then to TGF-β+AZD6738 treated sample. Mean signal shown for 5 biological replicates.

C) Violin plots showing average H2AK119ub1 enrichment via CUT&RUN-qPCR at *SNAI1* in the indicated cells treated for 24 h with 5 ng/mL TGF-β and 1 μM AZD6738. ATMi KU55933 (10 μM) was added at the same time as TGF-β and AZD6738. Signal is normalized to IgG and then to TGF-β+AZD6738 treated sample. Mean signal shown for 5 biological replicates.

D) Western blot showing SNAI1 and Actin protein levels in MCF10A pInducer RNaseH1-WT cells treated for 24 h with 5 ng/mL TGF-β and/or 1 μM AZD6738 and with either

100ng/mL Doxycycline to induce RNaseH1-WT expression or 10 μM of GSK126 or EPZ-6438.

E) Violin plots showing average H2AK119ub1 enrichment via CUT&RUN-qPCR at *SNAI1* shown in MCF10A cells treated for 24 h with 5 ng/mL TGF-β and 1 μM AZD6738 simultaneously and with either DMSO or 10 μM of GSK126 or EPZ-6438. Signal is normalized to IgG and then to TGF-β+AZD6738+DMSO treated sample. Mean signal shown for 3-4 biological replicates.

For **A-D,** 50-100ng/mL doxycycline was used to induce RNaseH1-WT or RNaseH1-D210N for 24 h prior treatment in indicated samples. Statistical analysis was done as in Figure 4.

Supplemental Figure 6. ATRi increases RNH-sensitive R loops and induces repressive chromatin at EMT genes after TGF-β treatment.

A) Plots of average R loop density at *ACTB* gene from DRIP-seq in MCF10A cells treated with DMSO, 5 ng/mL TGF-β, 1 μM AZD6738, or both for 24 h. TSS: Transcription start site, TTS: Transcription termination site, RNH: RnaseH.

B) Plots of average R loop density at EMT genes from DRIP-seq in MCF10A cells treated with 5 ng/mL TGF-β with and without RnaseH treatment. TSS: Transcription start site, TTS: Transcription termination site.

C) Plots of average R loop density at EMT genes from DRIP-seq in MCF10A cells treated with 5 ng/mL TGF-β and 1 μM AZD6738 with and without RNaseH treatment. TSS: Transcription start site, TTS: Transcription termination site.

D) Plots of average H2AK119ub1 density at EMT genes from CUT&TAG in MCF10A cells treated with either DMSO or 5 ng/mL TGF-β. TSS: Transcription start site, TTS: Transcription termination site.

E) Plots of average H2AK119ub1 density at EMT genes from CUT&TAG in MCF10A cells treated with 5 ng/mL TGF-β and/or 1 μM AZD6738 TSS: Transcription start site, TTS: Transcription termination site.

F) Gene tracks of EMT genes *SNAI1, MMP9,* and *SERPINE1* in indicated H2AK119ub1 CUT&TAG samples.

Statistical analysis was done as in Figure 5.

Supplemental Figure 7. ATR inhibition induces DNA double-stranded breaks during MET and suppresses tumor burden and metastasis

A) Quantification of nuclear γH2AX intensity (left) and representative images (right) in MDA-MB-231 cells treated with DMSO, 100 nM Eribulin, 1 μM AZD6738, or both for 24, 48, and 72 h. Mean integrated intensity with standard error of three biological replicates is shown. One-way ANOVA followed by Šídák's multiple comparisons test between Eribulin and Eribulin + AZD6738 was performed for all time points.

B) Line graph plotting primary tumor bioluminescence in indicated the cohorts from subcutaneous injection experiment in Figure 6C. Each value represents fold change in respective cohorts compared to measurements from week 1. Mean with standard error is shown for 3-4 mice in each group.

C) Line graph plotting primary tumor bioluminescence in indicated the cohorts from subcutaneous injection experiment in Figure 6C. Each value represents fold change

in respective cohorts compared to measurements from week 1. Mean with standard error is shown for 3-4 mice in each group. The difference in fold change at each timepoint in plotted below.

D) Line graph plotting primary tumor volume fold change in the indicated cohorts from orthotopic injection experiment in Figure 6C. Each value represents fold change in respective cohorts compared to measurements from week 1. Mean with standard error is shown for 3-4 mice in each group.

E) Line graph plotting primary tumor volume fold change in indicated cohorts from orthotopic injection experiment in Figure 6C. Each value represents fold change in respective cohorts compared to measurements from week 1. Mean with standard error is shown for 3-4 mice in each group. The difference in fold change at each timepoint in plotted below.

F) Representative image of H&E staining of lung tissue sections from orthotopic injection experiment in Figure 6C in indicated cohorts. Note that multiple metastases were detected in the same lung of a mouse induced to express SNAI1 and treated with vehicle.

Statistical analysis was done as in Figure 6.

Supplemental tables

Supplemental table 1: *SNAI1* qPCR primers for DRIP and CUT&RUN quantification.

Oligo Name	Sequence (5' to 3')
DRIP-1-F	CCGGAGTACTTAAGGGAGTTG
DRIP-1-R	CAGCAGTAGCGCAGAAGAA
DRIP-2-F	AGACGAGATGTGTGTGAGGA
DRIP-2-R	TTCCTGATCTCCCTCTCCTAAG
DRIP-3-F	CCTGGCTCTGAATCCTTCTTT
DRIP-3-R	GCTGTTCTCATGCCCATCT
DRIP-4-F	CGGGCTTTATTGGCATCTTATTC
DRIP-4-R	CAGGCAGTTGGACCTTTACA
DRIP-5-F	GGGTGAACTTACCCTTCTCATT
DRIP-5-R	CACTACTCCACTCTGCCATTAC
DRIP-6-F	GTAGCCTCTTGGGTGATAGAAAG
DRIP-6-R	CAATCAGGCTGAGTCCCATAAG
DRIP-7-F	CCACGAGGTGTGACTAACTATG
DRIP-7-R	ACCAAACAGGAGGCTGAAATA

Supplemental Methods

Cell culture

MCF10A parental and Z-cad sensor cells were cultured in DMEM/F12 (Invitrogen, 11330032) supplemented with 5% horse serum (Invitrogen, 16050122), 20 ng/mL EGF (Peprotech, AF-100-15), 0.5mg/mL hydrocortisone (Sigma, H-0888), 100 ng/mL Cholera toxin (Sigma, C-8052), 10ug/mL insulin (Sigma, I-1882), and penicillin-streptomycin (Invitrogen, 15070063). HEK293T, MDA-MB-231-LM, and MDA-MB-231 cells were cultured in DMEM (Invitrogen, 11965092) supplemented with 10% fetal bovine serum and 1% penicillin-streptomycin. BT549 and T47D cells were cultured in RPMI (Invitrogen, 11875135) supplemented with 10% fetal bovine serum and 1% penicillin-streptomycin. MCF10A Z-cad sensor cells were a gift from Jeffrey M. Rosen's lab at Baylor College of Medicine and MDA-MB-231-LM cells were a gift from Daniel Haber's lab at Massachusetts General Hospital. MCF10A, MDA-MB-231, HEK293T, BT549 and T47D cells were acquired from ATCC.

Generation of cell lines

Lentivirus was packaged using HEK293T cells. Cells were seeded in a 6-well plate at a density of 300,000 cells per well to achieve 30–50% confluency. For transfection, a mixture of 1 µg transfer plasmid, 0.75 µg PAX2 packaging plasmid, and 0.25 µg VSVG envelope plasmid was prepared in 100 µL Opti-MEM (Gibco) and combined with 5 µL of 1 mg/mL polyethylenimine (PEI). After 15 min of incubation at room temperature, 500 µL of fresh media was added to the transfection mix and applied to the cells following media aspiration. Cells were incubated for 3–4 h, after which the transfection media was replaced with fresh media. Viral supernatant was collected 48 h post-transfection, filtered

through a 0.45 μm filter, and supplemented with 8 μg/mL polybrene before adding to target cells. Cells were incubated overnight, followed by media change the next day. For stable selection, cells were treated with 1 μg/mL puromycin for 2–3 days or 200 μg/mL G418 for 4–7 days, depending on the selection marker, until control cells without virus died.

pInducer20 SNAIL1-S6A plasmid was generated by Shyamala Maheswaran's lab at MGH. pInducer20 RNASE H1-V5 was Topo-cloned using ppyCAG_RNaseH1_WT (Addgene, Plasmid #111906) into pENTR/d-topo using Topo kit (Thermo Fisher K240020) using following forward primer: 5'-CACCATGCCCAAGAAGAAGAG-3' and the following reverse primer: 5'-TTACGTAGAATCGAGACCGAG-3'.

RNAi transfection

For siRNA transfection, 5 μL of 20 μM siRNA stock (either siLuciferase (5′-CUUACGCUGAGUACUUCGATT-3′, Invitrogen), siATR (5'-CCUCCGUGAUGUUGCUUGATT-3'), or siATR-2 (ThermoFisher Scientific, s536 4390824) was added to an empty culture dish, resulting in a final concentration of 50 nM in 2 mL of media. Next, 5 μL of Lipofectamine RNAiMAX (Invitrogen) was mixed with the siRNA, followed by the addition of 500 μL Opti-MEM (Gibco). The mixture was incubated for 15 min at room temperature. Cells were then added to the dish, and the volume was brought to 2 mL with complete media. After 24 h, media was replaced with fresh, siRNA-free media. Experimental treatments were initiated 48 h post-siRNA transfection.

S9.6 antibody purification

Recombinant S9.6 antibody was purified from culture supernatants of HB8739 mouse monoclonal hybridoma cells (RGK60001). Purified IgG was reconstituted to a final

concentration of 1 mg/mL in 0.1 M PBS, pH 7.4, and aliquots were stored immediately at -20°C until use.

Immunofluorescence

All immunofluorescence experiments (except S9.6 IF, γH2AX in MDA-MB-231 and BT549) were performed by seeding cells on coverslips coated with Poly-L-lysine (Sigma, P4707). For EdU labeling, 10 mM EdU was added to cells 30 min prior to fixing. For RPA, γH2AX, and EdU staining, cells were washed once with PBS, then pre-extracted with cold CSK buffer (10 mM PIPES pH 7.0, 300 mM sucrose, 100 mM NaCl, 3 mM $MgCl_2$, 0.5% Triton X-100) for 5 min. After aspirating the CSK buffer, cells were fixed with 3% paraformaldehyde (PFA) and 2% sucrose for 15 min at room temperature, washed with PBS, and stored in PBS at 4°C until staining.

For all other antibodies (e.g., GFP, RFP, γH2AX in Z-cad cells), cells were washed once with PBS and immediately fixed with 3% PFA and 2% sucrose for 15 min at room temperature, without pre-extraction. After washing with PBS, cells were stored in PBS at 4°C.

For immunostaining, cells were permeabilized with PBS containing 0.5% Triton X-100 for 15 min at room temperature, blocked in 3% BSA in PBS with 0.5% Triton X-100 for 1 h, and then subjected to EdU or antibody staining. The following antibodies were used at the indicated dilutions: anti-γH2AX (1:1000, Millipore, 05-636), anti-RPA32 (1:1000, Abcam, ab2175), anti-RFP (1:1000, Chromotek, 5F8), Purified Mouse Anti-E-Cadherin Clone 36/E (1:1000, BD Biosciences, 610181), GFP Monoclonal Antibody (3E6)

(1:1000, Invitrogen, A-11120). Appropriate secondary antibodies from Jackson Immunoresearch were used at 1:10,000 dilution.

S9.6 immunofluorescence

Cells were seeded onto coverslips 24 h prior to treatment with DMSO, 5 ng/mL of TGF-β (R&D systems, 7754-BH-005) , 1 uM AZD6738 or both TGF-β and AZD6738. After 24 h, cells were fixed in 4% methanol-free formaldehyde for 10 min at room temperature. Following fixation, coverslips were washed 3 times with PBS for 5 min per wash. Next, cells were permeabilized using 0.1% Triton-X 100 in PBS for 10 min at room temperature and incubated in 1X RNaseH buffer with or without thermostable RNaseH1 (New England Biolabs, M0523S) overnight at 37°C. On the following day, cells were blocked using blocking buffer (5% goat serum, 0.1% Triton-X and 1% fish skin gelatin in PBS) for 1 h at room temperature and incubated in S9.6 antibody (1:100) in blocking buffer overnight. Coverslips were then washed with PBS + 0.25% Tween-20 3 times for 15 min per wash. Coverslips were then incubated in anti-mouse secondary antibody (1:500, Thermo Fisher Scientific, A-11001) for 1 h at room temperature in blocking buffer. Coverslips were then washed with PBS + 0.25% Tween-20 3 times for 15 min per wash, stained with DAPI (Invitrogen) for 5 min and mounted using Mowiol (Sigma-Aldrich). Cells were imaged using a Nikon 90i microscope. Nuclear S9.6 intensity was quantified on FIJI using a DAPI mask.

Where indicated, treatments with RNaseT1 (1:150, Thermo Fisher Scientific, EN0541) and RNaseIII (1:150, New England Biolabs, M0245S) were performed in 50 mM Tris-HCl (pH 7.6), 75 mM, KCl, 3 mM $MgCl_2$, 0.1% BSA for 2 h at 37°C prior to overnight treatment with RNaseH. Coverslips were processed and imaged as described above but with the

addition of anti-nucleolin antibody (1:1000, Abcam, ab22758) alongside S9.6 antibody and anti-rabbit secondary antibody (1:500, Thermo Fisher Scientific, A-11012) alongside anti-mouse secondary antibody (1:500, Thermo Fisher Scientific, A-11001). Cells were imaged using a Nikon 90i microscope. Nucleoplasmic S9.6 intensity was quantified on FIJI using a DAPI mask subtracting nucleolar S9.6 intensity using a nucleolin mask.

To prepare tissue sections, tumors were fixed in formalin for 48 hs, embedded in paraffin and sectioned. Slides were baked at 65°C for 30 min, incubated in 100% xylene for 10 min, and incubated in fresh 100% xylene for an additional 10 min to complete deparaffinization. Tissue sections were rehydrated by soaking in 100% ethanol twice, 95% ethanol, and 80% ethanol for 5 min each. Next, tissue sections were incubated in 70% ethanol, ddH_2O, and fresh ddH_2O for 10 min each. Antigen retrieval was performed by incubating slides in a 1% antigen unmasking solution with citric acid base (Vector laboratories, H-3300-250) and cooked in a pressure cooker on "high" for 30 min. The slides remained in the pressure cooker for an additional 30 min and then were allowed to cool to room temperature. Next, slides were placed in a 0.2% antigen unmasking solution under gentle agitation for 20 min. Tissue sections were incubated in 1X RNaseH buffer with or without thermostable RNaseH1 (New England Biolabs, M0523S) overnight at 37°C. On the following day, tissue sections were washed twice in PBS for 5 min and blocked using 1% goat serum, 0.5% Tween-20 and 1% fish skin gelatin in PBS for 1 h at room temperature and incubated in S9.6 antibody (1:100) in blocking solution overnight at 37°C. On the following day, slides were washed 3 times for 5 min each with 0.5% Tween-20 in PBS with gentle agitation. Coverslips were then incubated in anti-mouse secondary antibody (1:1000, Thermo Fisher Scientific, A-11001) for 1 h at room

temperature in blocking buffer. Coverslips were then washed twice with PBS + 0.5% Tween-20 for 5 min and washed twice with PBS for 5 min, stained with DAPI (Thermo Scientific, 62248) and mounted using SlowFade™ Diamond Antifade Mountant (Invitrogen, S36963). Tumors and RNaseH-treated controls were imaged using a Nikon 90i microscope. Nuclear S9.6 intensity was quantified on FIJI using a DAPI mask.

Proximity Ligation Assay

Cells were seeded on coverslips 24-48 hrs before drug treatment. RNH1-WT or RNH1-D210N-expressing cells were treated with 100 ng/mL doxycycline 24 hrs prior to treatment with AZD6738 and TFGβ. Cells on coverslips were then pre-extracted using cold 0.5% NP-40 (Sigma-Aldrich, I8896) for 3-5 min on ice and fixed with 4% paraformaldehyde for 15 min at room temperature. Coverslips were washed twice with PBS for 5 min and blocked with 2% BSA for 1 hr at room temperature. Cells were then incubated with 1:500 anti-RNAP II CTD (p-Ser2) antibody (Abcam ab5095) and 1:500 anti-PCNA antibody (Santa Cruz Biotechonologies FL261, #7907) overnight at 4°C. On the following day, cells were washed 3 times with PBS and incubated in a pre-mixed solution of PLA probe anti-mouse minus (Sigma-Aldrich DUO92004) and PLA probe anti-rabbit plus (Sigma-Aldrich DUO9200) for 1 h at 37°C. The Duolink In Situ Detection Reagents Red (Sigma-Aldrich DUO92008) were used to perform the PLA reaction according to manufacturer's protocol. The coverslips were stained with DAPI (Invitrogen) for 5 min and mounted using Mowiol (Sigma-Aldrich). Cells were imaged using a Nikon 90i microscope. Nuclear PLA foci was quantified on FIJI using a DAPI mask.

Transwell migration assay

Cells were cultured in modified MCF10A media containing 2% horse serum and no EGF for 24 h prior to seeding. For RNaseH1-WT and RNaseH1-D210N expressing MCF10A cells, 100 ng/mL of doxycycline (Millipore Sigma, D5207) was added 24 h prior to seeding. 75,000 cells were seeded on the insert of the Matrigel Invasion Chambers (Corning, 354480) in modified MCF10A media following rehydration based on the manufacturer's protocol. Complete MCF10A media was added in the wells. At the time of seeding, DMSO, 5 ng/mL of TGF-β (R&D systems, 7754-BH-005) , 1 μM AZD6738 (SelleckChem, S7693), or both were added to the insert. After 24 h, non-invading cells were removed by scrubbing with a cotton swab based on manufacturer's protocol. The invading cells were fixed in cold methanol for 5 mins and stained with 0.2% crystal violet. Inserts were rinsed in distilled water twice to remove excess crystal violet. Invading cells were imaged on Echo Revolve microscope at 10X. 5 random fields of view were imaged, and cell count was average per sample and normalized to DMSO.

Scratch Assay

T47D and BT549 cells were seeded in 6 well plates in RPMI+10% FBS. At 70-80% confluency, cells were washed once with PBS and RPMI+2% FBS (low serum media) was added. 16-24 h later, cells were scratched with a p200 pipette tip, washed with low serum media to remove scratched cells, and low serum media was added to each well alongside either DMSO, 5 ng/mL of TGF-β (R&D systems, 7754-BH-005) , 1 μM AZD6738 (SelleckChem, S7693), or both. Immediately after adding DMSO, TGF-β, and/or AZD6738, as well as 24 h after treatment, 3-5 images of the scratched region were taken for each experimental condition for each replicate. The Wound Healing Tool on ImageJ was used to determine percent wound closure and the following formula was

used to calculate percent invasion: ((Area 0h – Area 24h)/Area 0h) x 100 where Area 0h refers to wound area at 0 h and Area 24 h refers to wound area at 24 h.

Chromatin Fractionation

Cells were washed with ice-cold 1X PBS and lysed in 100 uL of Buffer A (10 mM HEPES, pH 7.5, 10 mM KCl, 1.5 mM MgCl2, 340mM Sucrose, 10% Glycerol, 1 mM DTT, 0.1% Triton X-100) for 5 min on ice. Cells were spun at 1400 g for 5 min and supernatant was collected as the cytoplasmic fraction. Isolated nuclear pellet was washed 2X with Buffer A and were lysed in 100 uL Buffer B (3 mM EDTA, 0.2 mM EGTA, 1 mM DTT) and incubated on ice for 30 min to get soluble nuclear proteins. Lysed nuclei were spun at 2000 g for 5 min and supernatant was collected as soluble nuclear fraction. Chromatin pellet was washed 2X with Buffer B and then resuspended in 100 uL Buffer A and sonicated for 30 sec ON, 1 min OFF protocol 3X. All fractions were mixed with SDS loading buffer and denatured at 95°C for 5 min.

Western blotting

Cells were pelleted and snap frozen in dry ice. Pellets were lysed on ice in cold CSK buffer (10 mM Pipes pH 7.0, 300 mM sucrose, 100 mM NaCl, 3 mM MgCl2) with 0.5% Triton X-100 and protease inhibitor cocktail, 1 μM sodium fluoride, 1 μM sodium orthovanadate and 0.5 U/uL of Benzonase (Millipore Sigma, E1014-5KU) for 20 min. 2x lysis buffer (2% SDS and 200 mM NaCl) was added and BCA assay was performed to determine protein concentration. 2X gel loading buffer was added and samples were incubated at 95°C for 5 min before running gels and transferring to PVDF membrane. Membranes were blocked in 5% milk and TBST for 1 h at room temperature. Membranes were incubated in primary antibody overnight in 2.5% milk in TBST at 4°C. On the

following day, membranes were washed 3 times for 10 min in TBST, and incubated in secondary antibody at 1:10,000 in 2.5% milk in TBST for 1 h at room temperature. Membranes were washed 3 times for 10 min in TBST and imaged on the chemidoc using ECL (Bio Rad, Cat 170-0561).

DNA-RNA Immunoprecipitation

DNA isolation and digestion

MCF10A cells were treated with TGF-β (R&D systems, 7754-BH-005) and/or AZD6738 (SelleckChem, S7693) for 24 h, pelleted, and resuspended in 1.6 mL of TE buffer (10 mM Tris (Thermo Fisher Scientific, AM9855G), pH 8; 1 mM EDTA (Thermo Fisher Scientific, AM9260G)). 50 µL of 20%(wt/vol) SDS (Sigma-Aldrich, 05030) and 5 µL of 20 mg/mL proteinase K (Roche, 03115828001) were added to the solution. Samples were gently inverted and incubated overnight at 37°C. On Day 2, DNA lysate was poured directly into 15 mL pelleted high-density Maxtract phase-lock gel tubes (Qiagen, 129065) and 1.6 mL of phenol/chloroform/isoamyl alcohol (25:24:1) (ThermoFisher Scientific, 15593031) was added. The tubes were inverted several times and spun at 1,500g for 5 min. 160 µL of 3M NaOAc, pH 5.2 (Sigma-Aldrich, S7899) and 4 mL of ethanol were added to a new 15 mL tube. The DNA was poured into the phase-lock gel tube and the solution was gently inverted until the DNA fully precipitated. The DNA was spooled out of the mixture with cut tips and transferred to a clean 2 mL tube. The spooled DNA was washed with 1.5 mL of 80% ethanol by inverting tubes three times and incubated at room temperature for 10 mins. The wash step was repeated twice, 80% ethanol was removed, and the DNA pellet was allowed to dry completely before adding 125 uL of TE to the pellet. The tubes were kept on ice for 1 hr and the DNA was gently resuspended with a cut 200 uL pipette tip.

The tube was left on ice for another h. 100 uL of genomic DNA was digested using BsrGI, EcoRI, HindIII, SspI and XbaI (New England BioLabs, R0575S, R0101S, R0104S, R0132S and R0145S, respectively) in NEBbuffer 2 (New England Biolabs, B7002S) with 1X BSA (New England BioLabs, B9001) and 0.5X spermidine (Sigma-Aldrich, 05292) overnight at 37°C. On Day 3, the digested DNA was transferred to pelleted 2 mL light phase-lock gel tubes (VWR, 10847-800). 100 µL of water and 250 µL of phenol/chloroform/isoamyl alcohol (25:24:1) was added, the tubes were inverted five times, and spun at 16,000g for 10 mins. The DNA was then transferred to a new 1.5 mL tube containing 1.5 µL of glycogen (Thermo Fisher Scientific, R0561), 25 µL of 3M NaOAc, pH 5.2, and 625 uL of 100% ethanol, inverted 5 times and incubated at -20°C for 1 hr. Tubes were spun at 16,000g for 35 mins at 4°C. The supernatant was discarded, the pellet was washed with 200 µL of ethanol, and spun for 16,000g for 10 min at 4°C. The pellet was airdried for 15-20 mins, resuspended in 50 uL of TE buffer, and incubated on ice for 1 hr. The concentration of DNA was measured (OD260) using a NanoDrop (Thermo Scientific).

<u>S9.6 DRIP</u>

For each sample, 10 ug of digested DNA was treated with 10 uL of RNaseH (New England BioLabs, M0297S) overnight at 37°C. On Day 4, 10 ug of digested DNA not treated with RnaseH (two tubes per sample) as well as 10 ug of RNaseH-treated DNA was diluted in 500 uL of TE and 50 uL was stored for downstream qPCR applications. 52 µL of 10x DRIP binding buffer (100 mM sodium phosphate, pH 7, 1.4 M NaCl and 0.5% (vol/vol) Triton X-100) and 20 µL of the S9.6 antibody) were added to the remaining 450 µL in each tube and incubated overnight at 4°C in a mini-tube rotator. On Day 5, 40 uL of Pierce

protein A/G magnetic beads slurry (ThermoFisher Scientific, 88803) per tube was washed with 700 uL of 1x DRIP binding by inverting the tubes on a mini-tube rotator for 10 min at room temperature. The beads were isolated using a DynaMag-2 magnet (ThermoFisher Scientific, 12321D) and supernatant was discarded. This wash step was repeated twice. The S9.6:gDNA solution was then added to the washed beads and incubated at 4°C for 2 h while gently rotating on a mini-tube rotator. Next, the beads were isolated using a DynaMag-2 magnet and supernatant was discarded. 750 µL of 1x DRIP binding buffer was added to each of the tubes which were then inverted for 15 mins at room temperature. The beads were isolated again and the wash step was repeated once more. Next, 300 µL of DRIP elution buffer (50 mM Tris, pH 8.0, 10 mM EDTA, pH 8.0, and 0.5% (vol/ vol) SDS) and 7 µL of proteinase K (20 mg/mL) was added to the beads and the tubes incubated at 55°C for 45 mins, gently vortexing the tubes every 10 mins. The beads were isolated for the final time and the supernatant was transferred to pelleted 2 mL light phase-lock gel tubes and spun at 16,000g for 1 min. 300 µL of phenol/chloroform/isoamyl alcohol (25:24:1) was added to each tube, which were gently inverted and spun at 16,000g for 10 mins at room temperature. The top layer was then transferred to a new 1.5 mL tube containing 1.5 µL of glycogen, 25 µL of 3M NaOAc, pH 5.2, and 625 uL of 100% ethanol, inverted 5 times and incubated at -20°C for 1 h. Tubes were spun at 16,000g for 35 mins at 4°C. The supernatant was discarded, the pellet was washed with 200 µL of ethanol, and spun for 16,000g for 10 min at 4°C. The pellet was airdried for 10-15 mins. Tubes containing samples pre-treated with RNaseH were resuspended in 50 uL of TE buffer and incubated on ice for 30 mins. Tubes containing samples not previously treated with RNaseH (2 tubes per sample) were resuspended in 25 uL of TE buffer, incubated on ice

for 30 mins, and then combined into one 50 uL tube. qPCR was used to quantify pull-downs at various loci. Primer sequences are reported in Supplementary Table 1.

DRIP-seq library preparation

DRIP for library preparation was performed using 10 ug of digested DNA as well as 10 ug of RNaseH-treated DNA, following protocol outlined above. Library preparation was performed using NEBNext® Multiplex Oligos for Illumina® (Index Primers Set 1) (NEB, E7335S) following manufacturer's instructions.

DRIP-seq analysis

The raw FASTQ reads were processed using Trim Galore (v0.6.6) to remove Illumina adapters. The trimmed reads were then mapped to the human genome (Gencode v42) using BWA (v0.7.15). Duplicate reads were removed using Samtools (v1.4.1). The bamCoverage command from deepTools (v3.5.1) was used to compute read coverage across genomic regions from the BAM files, generating the corresponding bigWig files.

The TGF-β -induced EMT gene set was defined based on RNA-seq data from GSE210499, selecting genes with a log2 fold change > 0.5 and p-value < 0.05 after 24 h of treatment compared to the control. All other protein-coding genes, excluding those in the EMT gene set, were categorized as the "other gene set." The R package ggChIPvis (available at https://github.com/junjunlab/ggChIPvis) was used to generate profile plots for different genomic regions across various samples.

CUT&RUN

<u>Cell treatment and preparation</u>

MCF10A cells were treated with either DMSO, 5 ng/mL of TGF-β, 1 μM AZD6738 or both TGF-β and AZD6738 for 24 h. For RNaseH1-WT and RNaseH1-D210N expressing MCF10A cells, 100 ng/mL of doxycycline (Millipore Sigma, D5207) was added 24 h prior to treatment with TGF-β and/or AZD6738. For each sample, $3x10^5$ cells were harvested and washed twice in ice-cold Wash Buffer (20 mM HEPES-KOH, pH 7.5, 150 mM NaCl, 0.5 mM Spermidine, protease inhibitors). After the final wash, cells were resuspended in 200 μL of Wash Buffer per reaction and kept on ice until further processing.

Preparation of Con A-coated beads

BioMag® Plus Concanavalin A-coated magnetic beads (10 uL/reaction) were activated by washing twice with 1.5 mL of Binding Buffer (20 mM HEPES-KOH, pH 7.9, 10 mM KCl, 1 mM $CaCl_2$, 1 mM $MnCl_2$) and resuspended to the original bead slurry volume (10 μL per reaction). For each reaction, 10 μL of bead slurry was added to 200 μL of cells ($3x10^5$ cells per reaction) and incubated at room temperature for 10 mins with gentle rotation to allow binding of cells to the beads.

Primary Antibody Binding

The bead-bound cells were washed twice with 1.5 mL of Wash Buffer and resuspended in 200 μL of Antibody Buffer (Wash Buffer containing 4 uL/mL of 0.5M EDTA and 0.0125% Digitonin (EMD Millipore, 300410). Anti-IgG antibody, anti-H2AK119ub1 antibody (Diagenode, C15410002), or anti-γH2AX antibody (Millipore Sigma, 05-636) were added at a dilution of 1:100 in Antibody Buffer and incubated overnight at 4°C with rotation.

Binding of pAG-MNase

Following primary antibody incubation, cells were washed twice in 1.5 mL of Digitonin Wash Buffer (0.0125% Digitonin in Wash Buffer). 2 uL/reaction of pAG-MNase (Cell Signaling Technology, 40366S) in Dig-Wash Buffer was added to the cell-bead complexes (200 μL per reaction). The mixture was incubated at 4°C for 2 h with gentle rotation to allow binding of the pAG-MNase to the antibody-protein complexes.

Chromatin Digestion

After pAG-MNase binding, cells were chilled on ice for 5 min. Chromatin digestion was initiated by adding 3 μL of 100 mM $CaCl_2$ to each reaction while vortexing, and reactions were incubated on ice for 30 min to allow chromatin cleavage by MNase. The digestion was stopped by adding 150 μL of 2xSTOP Buffer (200 mM NaCl, 20 mM EDTA, 4 mM EGTA, 0.05% Digitonin, 50 μg/mL RNase A (Thermo Fisher Scientific, EN0531), 40 μg/mL glycogen (Sigma-Aldrich, 10901393001)).

Chromatin Fragment Isolation

Reactions were incubated at 37°C for 10 min to release soluble chromatin fragments. After incubation, samples were centrifuged at 16,000 x g for 5 min at 4°C, and the supernatant containing the chromatin fragments was transferred to a fresh 1.5 mL microcentrifuge tube.

DNA Extraction and qPCR

DNA was extracted by adding 3 μL of 10% SDS and 2.5 μL of Proteinase K (20 mg/mL, New England BioLabs, P8107S) to each sample, followed by incubation at 70°C for 10 min. After cooling, 300 μL of phenol-chloroform-isoamyl (25:24:1) was added to each sample, vortexed for 30 sec, and centrifuged at 16,000 x g for 5 min at room temperature.

The aqueous phase was transferred to a phase-lock gel tube and extracted again with 300 µL of chloroform. After centrifugation, the aqueous phase was transferred to a new tube containing 2 µL of 2 mg/mL glycogen. DNA was precipitated with 750 µL of 100% ethanol, chilled on ice for 10 min, and centrifuged at 16,000 x g for 10 min at 4°C. The DNA pellet was washed twice with 1 mL of 100% ethanol, air-dried, and resuspended in 100 µL of sterile water. Samples were run on a 384-well plate using Applied Biosystems™ PowerUp™ SYBR™ Green Master Mix for qPCR (Fisher Scientific, A25742) on the LightCycler® 480 Instrument II using SNAI1 DRIP primers.

RNA isolation and quantitative PCR

RNA was prepared from MCF10A cells treated with DMSO, TGF-β, AZD6738, or both TGF-β and AZD6738 for 24 h with TRIzol Reagent (Invitrogen, 15596026) using manufacturer's protocol and quantified using NanoDrop. cDNA was prepared using SuperScript™ IV First-Strand Synthesis System (Invitrogen, 18091050) using manufacturer's instructions and run on a 384-well plate using Applied Biosystems™ PowerUp™ SYBR™ Green Master Mix for qPCR (Fisher Scientific, A25742) on the LightCycler® 480 Instrument II using the following primers for SNAI1: forward: 5′-GCTGCAGGACTCTAATCCAGA-3′, reverse: 5′-ATCTCCGGAGGTGGGATG-3′ and GAPDH: forward: 5′-AGCCACATCGCTCAGACA-3′, reverse: 5′-TGGACTCCACGACGTACT-3′.

H2AK119ub1 CUT&TAG

MCF10A cells were seeded and treated as described in the CUT&RUN section above. 5 x 10^5 cells washed twice with 1.0 mL of wash buffer (20 mM HEPES (pH 7.5), 150 mM

NaCl, 0.5 mM spermidine, and 1x protease inhibitors). Concanavalin A-coated magnetic beads (10 μl/reaction) were activated and incubated with the cells at room temperature for 10 mins. After removing the supernatant, the bead-bound cells were resuspended in 100 μl of antibody buffer (20 mM HEPES (pH 7.5), 150 mM NaCl, 0.5 mM spermidine, 1X protease inhibitors, 0.05% digitonin, 0.01% NP-40, and 2 mM EDTA). Subsequently, 1.65 ug H2AK119ub antibody (Diagenode, C15410002) was added, and the mixture was incubated overnight at 4°C with rotation.

After two washes with dig-wash buffer (20 mM HEPES (pH 7.5), 150 mM NaCl, 0.5 mM spermidine, 1x protease inhibitors, 0.05% digitonin, 0.01% NP-40), the samples were incubated with 1.5ug mouse anti-rabbit IgG (Sigma, R2655) in 100 μl of antibody buffer at room temperature for 1 h. Bead-bound cells were washed once with 150 μl of dig-wash buffer to remove unbound antibodies. Secondary rabbit anti-mouse IgG antibody (1.5ug; Thermo Scientific, 31188) diluted 1:100 incubated for another hour, followed by three washes in dig-wash buffer.

The pA-Tn5 adapter complex (Cell Signaling Technology, 79561) was diluted 1:25 in dig-300 buffer (20 mM HEPES (pH 7.5), 300 mM NaCl, 0.5 mM spermidine, 1X protease inhibitors, 0.05% digitonin), and with the bead-bound cells with 50 μl diluted pA-Tn5 incubated at room temperature for 1 h with rotation. Cells were then washed three times in dig-300 buffer to remove unbound pA-Tn5. Tagmentation was performed by resuspending cells in 40 μl of tagmentation buffer (10 mM TAPS-KOH (pH 8.3), 10 mM $MgCl_2$, 10% DMF) and incubating at 37°C for 1 h. The reaction was stopped by adding

1.5 µl of 0.5 M EDTA, 0.5 µl of 10% SDS, and 1 µl of proteinase K (20 mg/ml; Roche), followed by incubation at 55°C for 1 h and 75 °C for 20 min.

Tagmentation products were purified with 1.8x AMPure XP beads (Beckman,20745200) and eluted in 10 µl of 0.1% Tween 20. The eluent was mixed with 10 U of Bst 2.0 WarmStart DNA polymerase (NEB, M0538) and 0.4 U Q5 high-fidelity DNA polymerase (NEB, M0491L) in 1x Q5 polymerase reaction buffer, incubated at 65°C for 30 minutes to conduct the strand displacement reaction, and heat-inactivated at 80°C for 20 minutes. Sequencing libraries were generated by amplifying with a universal i5 primer and a unique i7 primer using Q5 high-fidelity DNA polymerase. Libraries were size-selected with 0.5x SPRIselect beads (Beckman, B23318) and validated by Agilent TapeStation analysis before Illumina sequencing.

CUT&TAG Data Analysis

The data analysis workflow was similar to DRIP-seq, except that Bowtie2 (v2.5.4) was used for mapping instead of BWA.

Xenotransplantation and Bioluminescence Imaging

For the subcutaneous injection experiment, 500,000 BT549 cells transduced with pLenti-CMV-Luciferase (Addgene, 17477) expressing either pInducer-empty vector or pInducer-SNAI1 were injected subcutaneously into flanks of NSG mice (Charles River) in 50% PBS and 50% Matrigel Matrix (Corning Life Sciences). After four weeks, mice were administered doxycycline in water with sucrose for 5 consecutive days alongside either DMSO or ATRi (AZD6738; 50 mg/kg) via oral gavage. 6 mice (3 mice injected with BT549-EV and 3 mice injected with BT549-SNAI1) treated with Dox and ATRi were sacrificed

and the tumors were fixed in formalin and embedded in paraffin for downstream applications. 4 mice in each of the remaining cohorts (BT549-EV+DMSO, BT549-EV+ATRi, BT549-SNAI1+DMSO, BT549-SNAI1+ATRi) were monitored every 2 weeks using bioluminescence imaging.

For the orthotopic injection experiment, 750,000 BT549 cells transduced with pLenti-CMV-Luciferase (Addgene, 17477) expressing either pInducer-empty vector or pInducer-SNAI1 were injected into the mammary fat pads NCG mice (Charles River) in 50% PBS and 50% Matrigel Matrix (Corning Life Sciences). After 2 weeks, mice were administered doxycycline in water with sucrose for 5 consecutive days alongside either DMSO or ATRi (AZD6738; 50 mg/kg) via oral gavage. 4 mice in each of the cohorts (BT549-EV+DMSO, BT549-EV+ATRi, BT549-SNAI1+DMSO, BT549-SNAI1+ATRi) were monitored every 2 weeks using bioluminescence imaging. Primary tumor volume measurements were also taken every Monday and Thursday for the duration of the study. One mouse in the BT549-SNAI1+DMSO cohort was found dead, and tissue could not be collected for additional staining. On Day 46, the remaining mice were sacrificed and examined for metastatic lesions. Tumor and additional tissue including lung, liver, mammary fat pad, and spleen, were collected, fixed in formalin, and embedded in paraffin prior to tissue sectioning and H&E staining.

For experiments using MDA-MB-231-LM-Luciferase cells, 200,000 cells were injected into the tail vein of each mouse (4 mice per cohort). Mice were administered DMSO or 50 mg/kg of ATRi (AZD6738) on a 5-day on 2-day off schedule. Mice were treated for 5 weeks with week 3 off and monitored every week using bioluminescence imaging.

IVISbrite D-Luciferin Bioluminescent Substrate in RediJect Solution (XenoLight) (Perkin Elmer, 770504) was thawed in accordance with the manufacturer's instructions. 150 uL of this substrate was injected intraperitoneally into each mice. Mice were imaged 5 min after administration. Isoflurane was set to 2.5% with oxygen. The IVIS flow was set to 1 and chamber flow to 2. Exposure time was set to automatic for image acquisition. Luciferase intensity was analyzed using LivingImage 4.0 Software (Caliper Life Sciences).

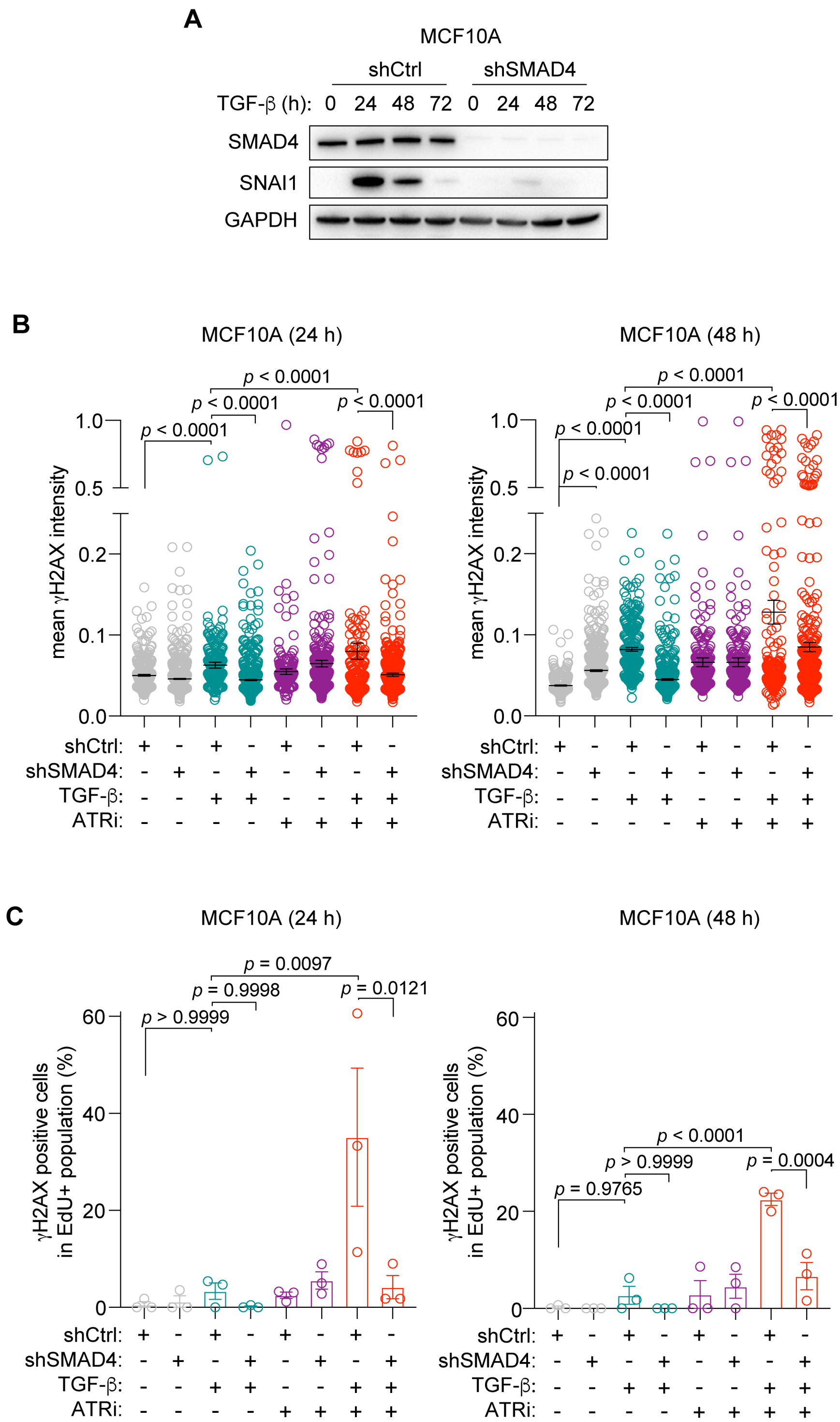
A
MCF10A
shCtrl
shSMAD4
TGF-β (h): 0 24 48 72 0 24 48 72
SMAD4
SNAI1
GAPDH
B
MCF10A (24 h)
MCF10A (48 h)
p < 0.0001
mean γH2AX intensity
1.0
0.5
0.2
0.1
0.0
shCtrl: + - + - + - + -
shSMAD4: - + - + - + - +
TGF-β: - - + + - - + +
ATRi: - - - - + + + +
C
MCF10A (24 h)
MCF10A (48 h)
p > 0.9999
p = 0.9998
p = 0.0097
p = 0.0121
p = 0.9765
p > 0.9999
p < 0.0001
p = 0.0004
γH2AX positive cells in EdU+ population (%)
60
40
20
0
shCtrl: + - + - + - + -
shSMAD4: - + - + - + - +
TGF-β: - - + + - - + +
ATRi: - - - - + + + +

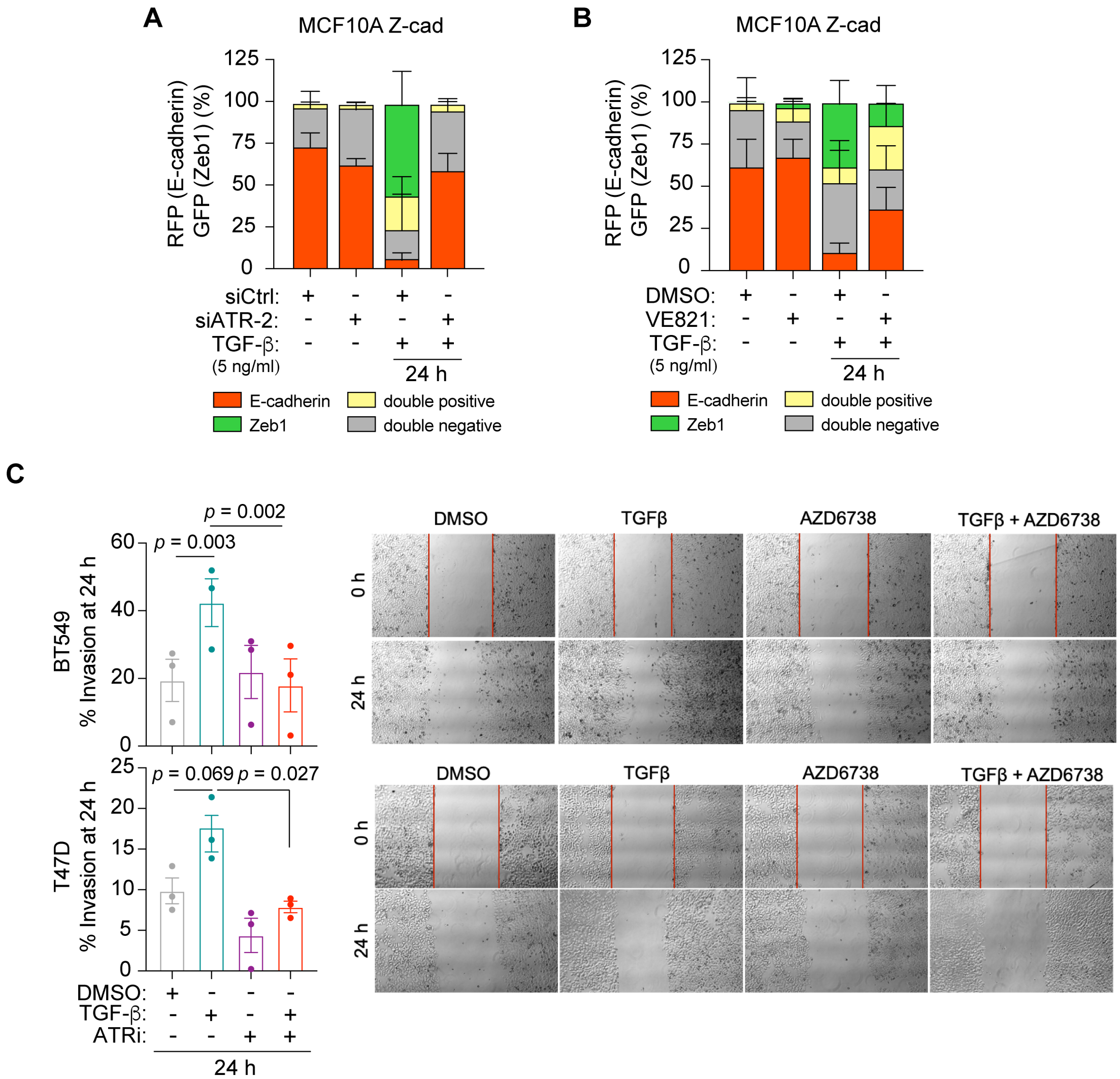
A
MCF10A Z-cad
RFP (E-cadherin)
GFP (Zeb1) (%)
siCtrl:
siATR-2:
TGF-β:
(5 ng/ml)
24 h
E-cadherin
Zeb1
double positive
double negative
B
MCF10A Z-cad
DMSO:
VE821:
TGF-β:
(5 ng/ml)
24 h
C
BT549
% Invasion at 24 h
p = 0.003
p = 0.002
T47D
% Invasion at 24 h
p = 0.069
p = 0.027
DMSO:
TGF-β:
ATRi:
24 h
DMSO
TGFβ
AZD6738
TGFβ + AZD6738
0 h
24 h

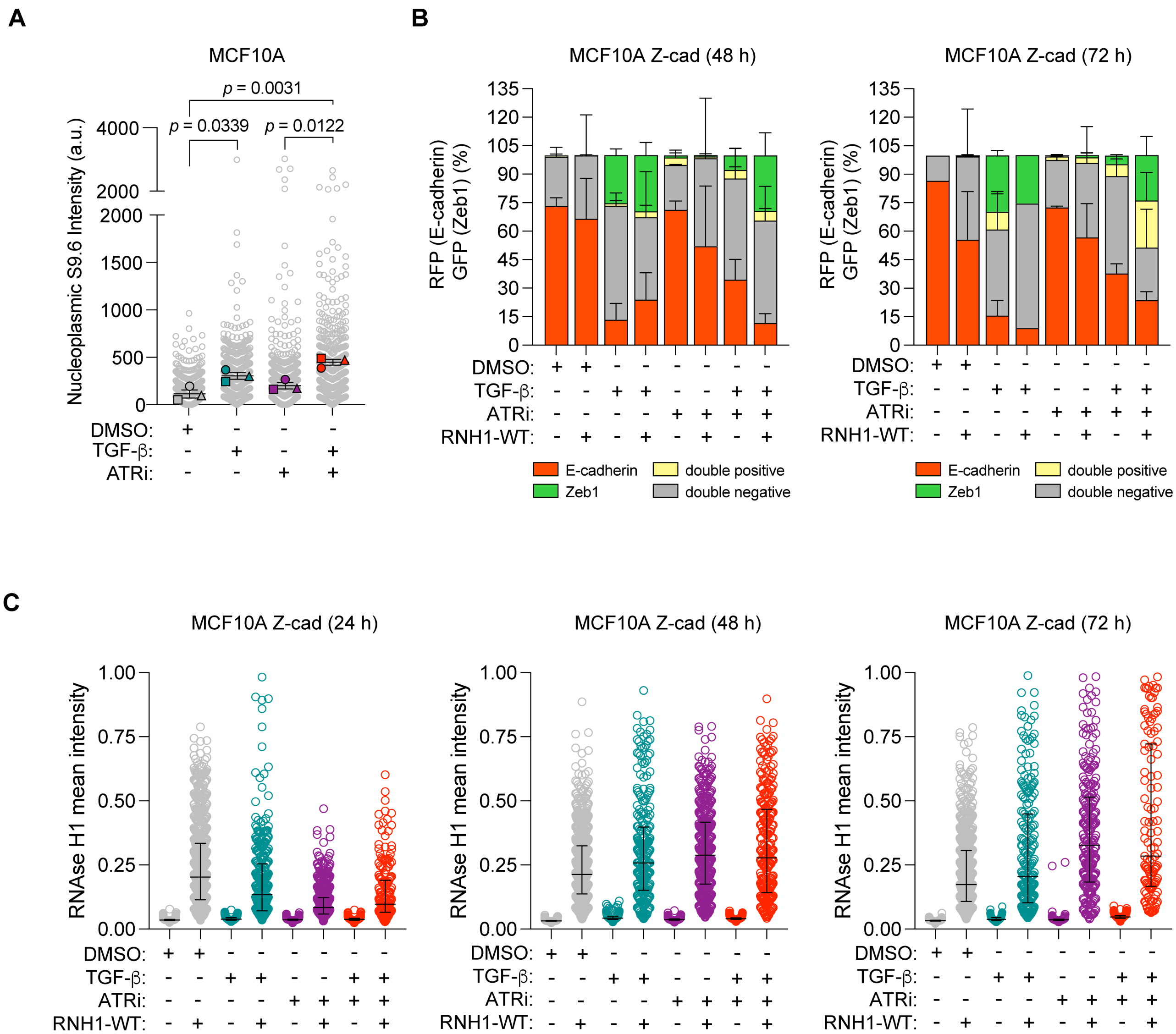

A
MCF10A
p = 0.0031
p = 0.0339
p = 0.0122
Nucleoplasmic S9.6 Intensity (a.u.)
4000
2000
2000
1500
1000
500
0
DMSO: + - - -
TGF-β: - + - +
ATRi: - - + +
B
MCF10A Z-cad (48 h)
MCF10A Z-cad (72 h)
RFP (E-cadherin)
GFP (Zeb1) (%)
135
120
105
90
75
60
45
30
15
0
DMSO: + + - - - - - -
TGF-β: - - + + - - + +
ATRi: - - - - + + + +
RNH1-WT: - + - + - + - +
E-cadherin
double positive
Zeb1
double negative
C
MCF10A Z-cad (24 h)
MCF10A Z-cad (48 h)
MCF10A Z-cad (72 h)
RNAse H1 mean intensity
1.00
0.75
0.50
0.25
0.00
DMSO: + + - - - - - -
TGF-β: - - + + - - + +
ATRi: - - - - + + + +
RNH1-WT: - + - + - + - +

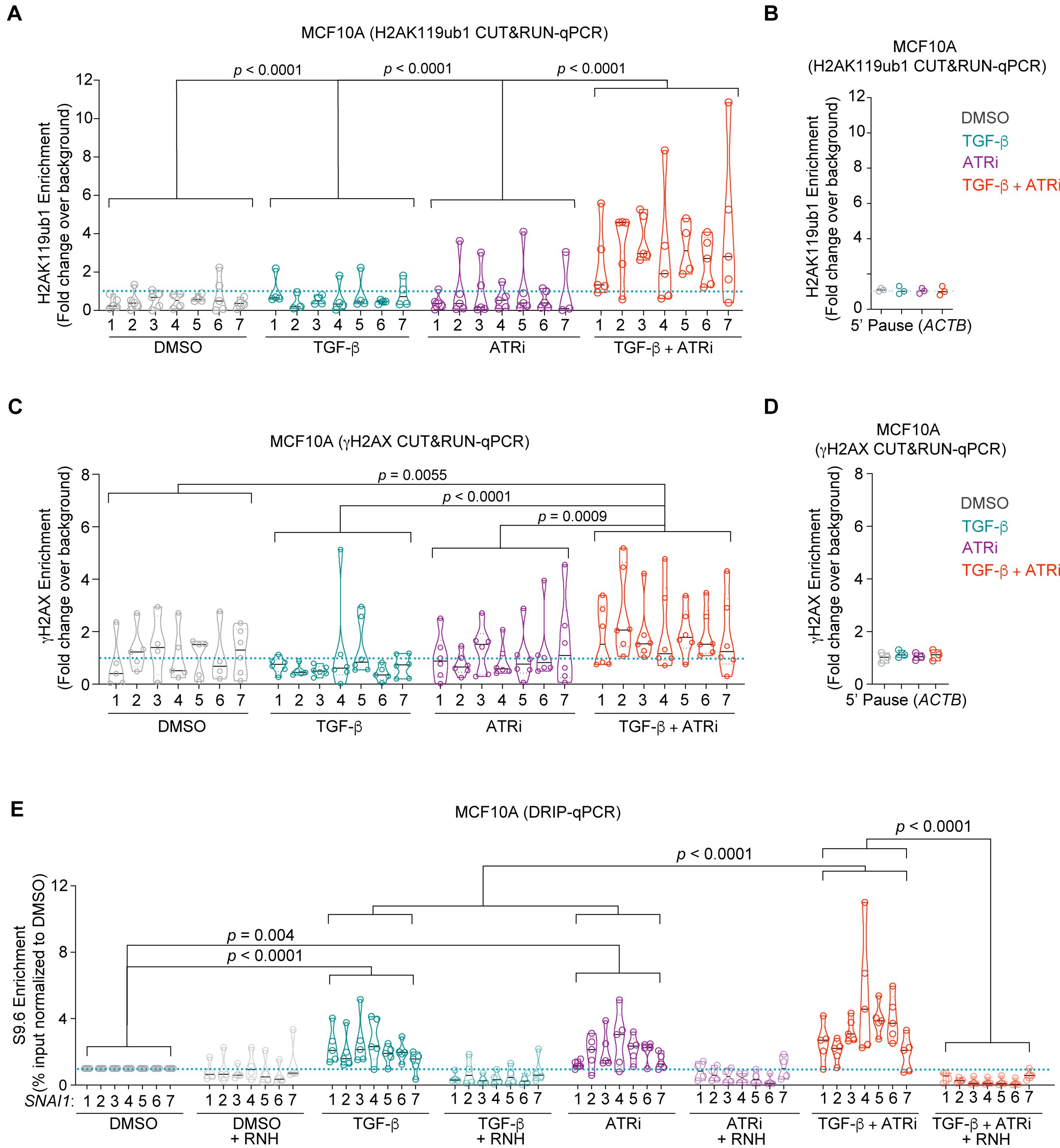
A
MCF10A (H2AK119ub1 CUT&RUN-qPCR)
p < 0.0001
p < 0.0001
p < 0.0001
H2AK119ub1 Enrichment
(Fold change over background)
1 2 3 4 5 6 7
DMSO
TGF-β
ATRi
TGF-β + ATRi
B
MCF10A
(H2AK119ub1 CUT&RUN-qPCR)
DMSO
TGF-β
ATRi
TGF-β + ATRi
5' Pause (ACTB)
C
MCF10A (γH2AX CUT&RUN-qPCR)
p = 0.0055
p < 0.0001
p = 0.0009
γH2AX Enrichment
(Fold change over background)
D
MCF10A
(γH2AX CUT&RUN-qPCR)
E
MCF10A (DRIP-qPCR)
p < 0.0001
p < 0.0001
p = 0.004
p < 0.0001
S9.6 Enrichment
(% input normalized to DMSO)
SNAI1:
DMSO
DMSO + RNH
TGF-β
TGF-β + RNH
ATRi
ATRi + RNH
TGF-β + ATRi
TGF-β + ATRi + RNH

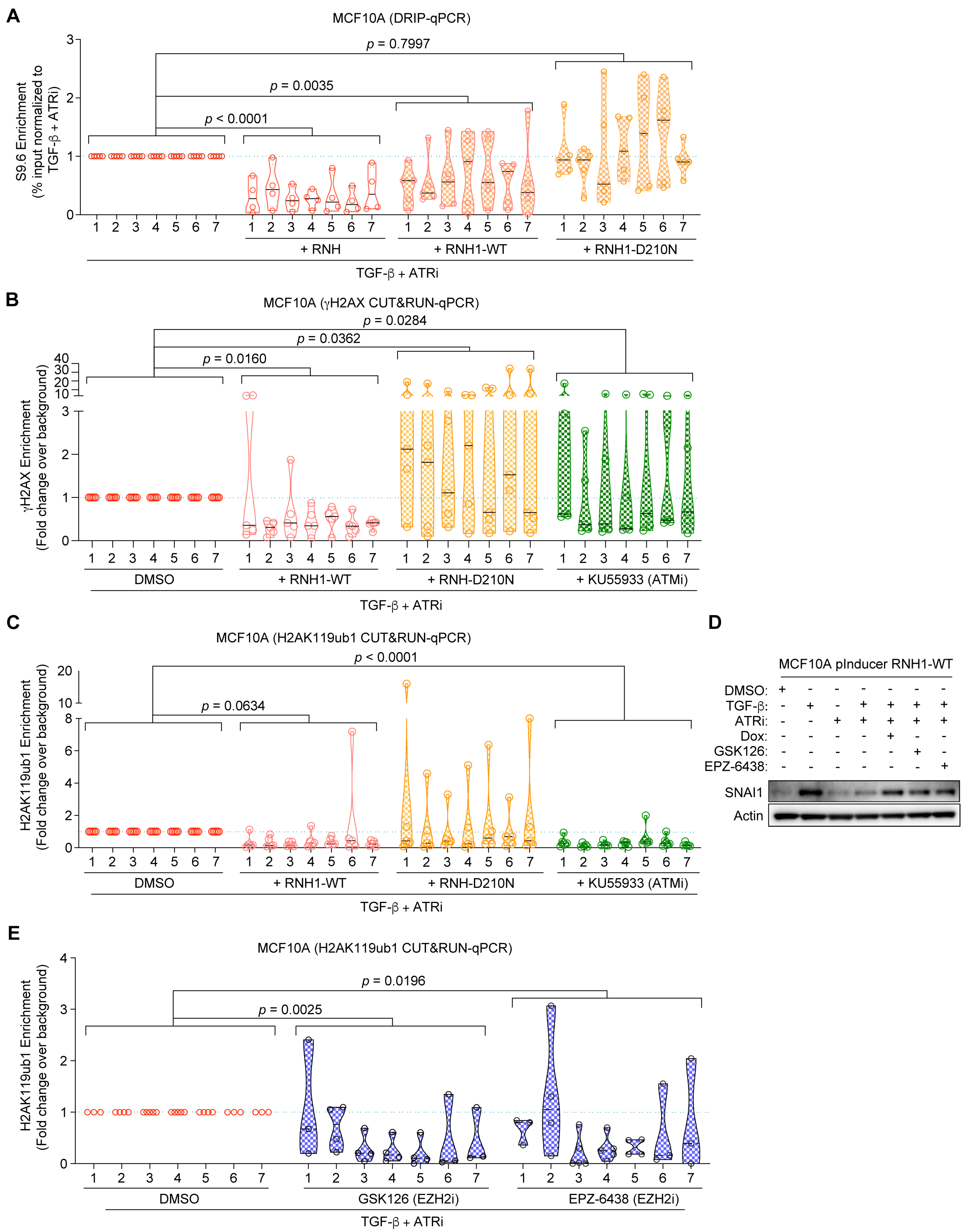

A
MCF10A (DRIP-qPCR)
S9.6 Enrichment (% input normalized to TGF-β + ATRi)
p = 0.7997
p = 0.0035
p < 0.0001
+ RNH
+ RNH1-WT
+ RNH1-D210N
TGF-β + ATRi
B
MCF10A (γH2AX CUT&RUN-qPCR)
γH2AX Enrichment (Fold change over background)
p = 0.0284
p = 0.0362
p = 0.0160
DMSO
+ RNH1-WT
+ RNH-D210N
+ KU55933 (ATMi)
TGF-β + ATRi
C
MCF10A (H2AK119ub1 CUT&RUN-qPCR)
H2AK119ub1 Enrichment (Fold change over background)
p < 0.0001
p = 0.0634
DMSO
+ RNH1-WT
+ RNH-D210N
+ KU55933 (ATMi)
TGF-β + ATRi
D
MCF10A pInducer RNH1-WT
DMSO:
TGF-β:
ATRi:
Dox:
GSK126:
EPZ-6438:
SNAI1
Actin
E
MCF10A (H2AK119ub1 CUT&RUN-qPCR)
H2AK119ub1 Enrichment (Fold change over background)
p = 0.0196
p = 0.0025
DMSO
GSK126 (EZH2i)
EPZ-6438 (EZH2i)
TGF-β + ATRi

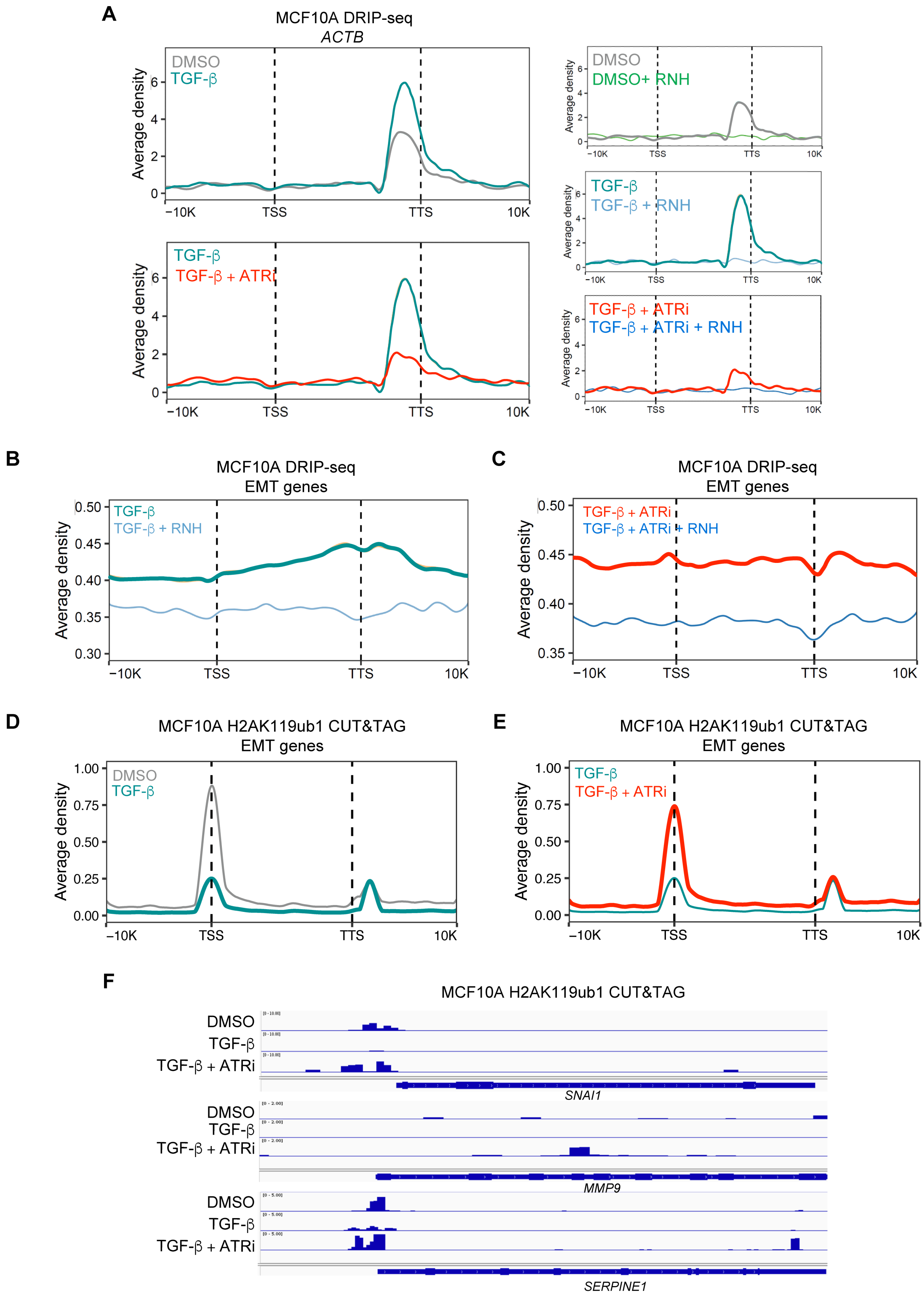
A
MCF10A DRIP-seq
ACTB
DMSO
TGF-β
Average density
TGF-β
TGF-β + ATRi
DMSO
DMSO+ RNH
TGF-β
TGF-β + RNH
TGF-β + ATRi
TGF-β + ATRi + RNH
−10K
TSS
TTS
10K
B
MCF10A DRIP-seq
EMT genes
TGF-β
TGF-β + RNH
C
MCF10A DRIP-seq
EMT genes
TGF-β + ATRi
TGF-β + ATRi + RNH
D
MCF10A H2AK119ub1 CUT&TAG
EMT genes
DMSO
TGF-β
E
MCF10A H2AK119ub1 CUT&TAG
EMT genes
TGF-β
TGF-β + ATRi
F
MCF10A H2AK119ub1 CUT&TAG
DMSO
TGF-β
TGF-β + ATRi
SNAI1
MMP9
SERPINE1

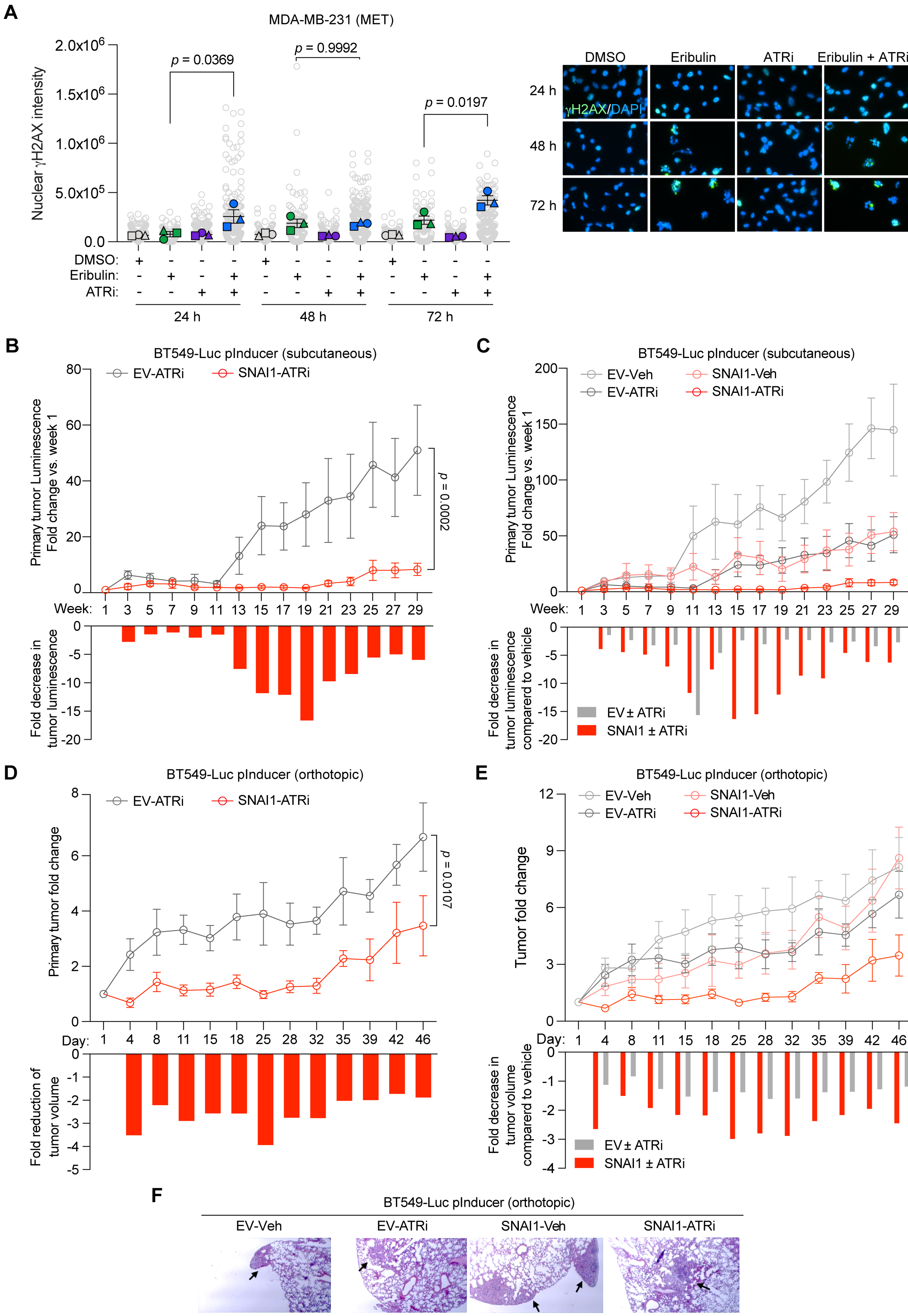
A
MDA-MB-231 (MET)
Nuclear γH2AX intensity
2.0x10^6
1.5x10^6
1.0x10^6
5.0x10^5
0.0
p = 0.0369
p = 0.9992
p = 0.0197
DMSO:
Eribulin:
ATRi:
24 h
48 h
72 h
DMSO
Eribulin
ATRi
Eribulin + ATRi
γH2AX/DAPI
B
BT549-Luc pInducer (subcutaneous)
EV-ATRi
SNAI1-ATRi
Primary tumor Luminescence Fold change vs. week 1
p = 0.0002
Week: 1 3 5 7 9 11 13 15 17 19 21 23 25 27 29
Fold decrease in tumor luminescence
C
BT549-Luc pInducer (subcutaneous)
EV-Veh
EV-ATRi
SNAI1-Veh
SNAI1-ATRi
Primary tumor Luminescence Fold change vs. week 1
Week: 1 3 5 7 9 11 13 15 17 19 21 23 25 27 29
Fold decrease in tumor luminescence compared to vehicle
EV ± ATRi
SNAI1 ± ATRi
D
BT549-Luc pInducer (orthotopic)
EV-ATRi
SNAI1-ATRi
Primary tumor fold change
p = 0.0107
Day: 1 4 8 11 15 18 25 28 32 35 39 42 46
Fold reduction of tumor volume
E
BT549-Luc pInducer (orthotopic)
EV-Veh
EV-ATRi
SNAI1-Veh
SNAI1-ATRi
Tumor fold change
Day: 1 4 8 11 15 18 25 28 32 35 39 42 46
Fold decrease in tumor volume compared to vehicle
EV ± ATRi
SNAI1 ± ATRi
F
BT549-Luc pInducer (orthotopic)
EV-Veh
EV-ATRi
SNAI1-Veh
SNAI1-ATRi
