# Supplementary material for: ATR safeguards epithelial-to-mesenchymal transition by countering R-loops and enabling transcription reprogramming
Source: J Clin Invest. 2026 Jan 22;136(16):e192225. doi: 10.1172/JCI192225 (PMC13476143; doi:10.1172/JCI192225)

Fully unedited blot/gel for Figure 1B

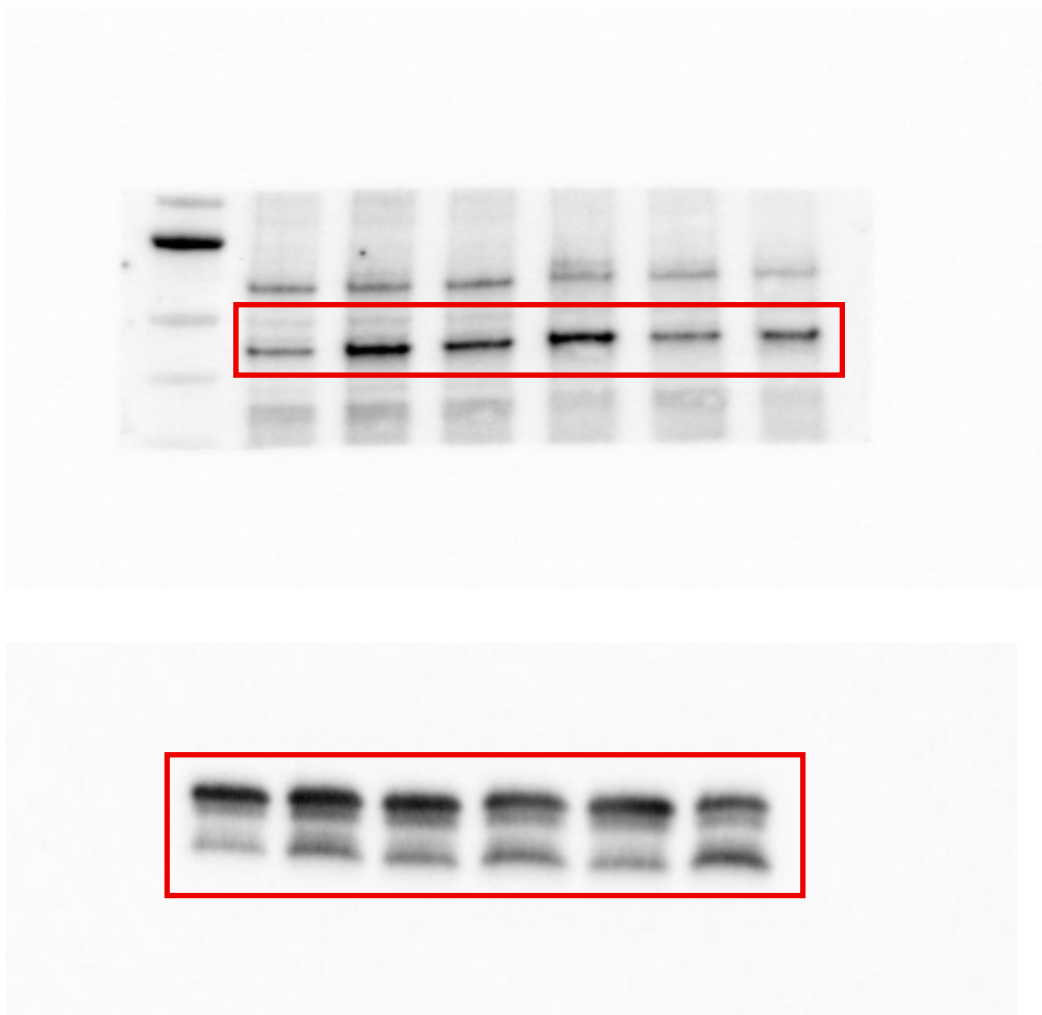

Fully unedited blot/gel for Figure 2A

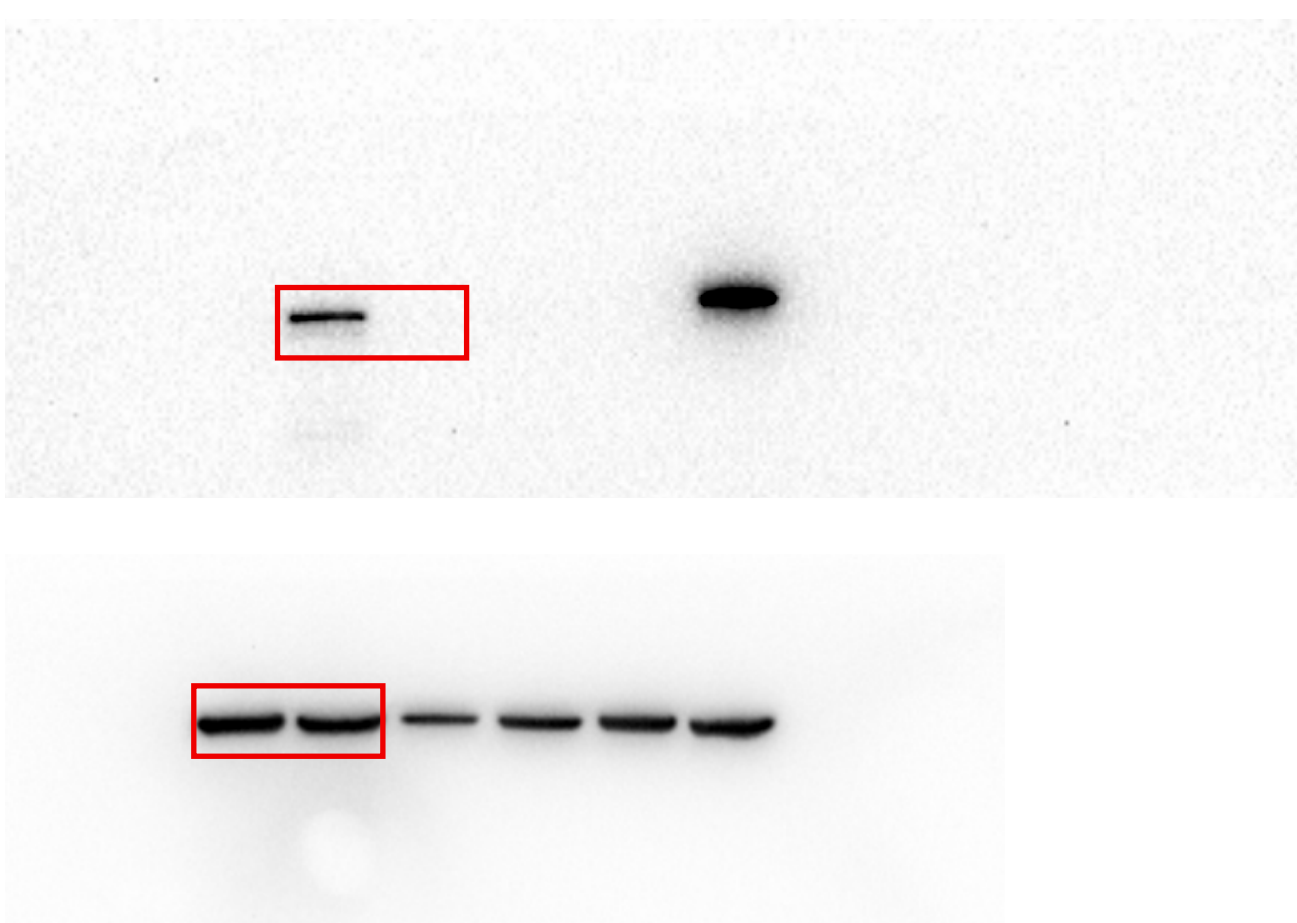

Fully unedited blot/gel for Figure 3I

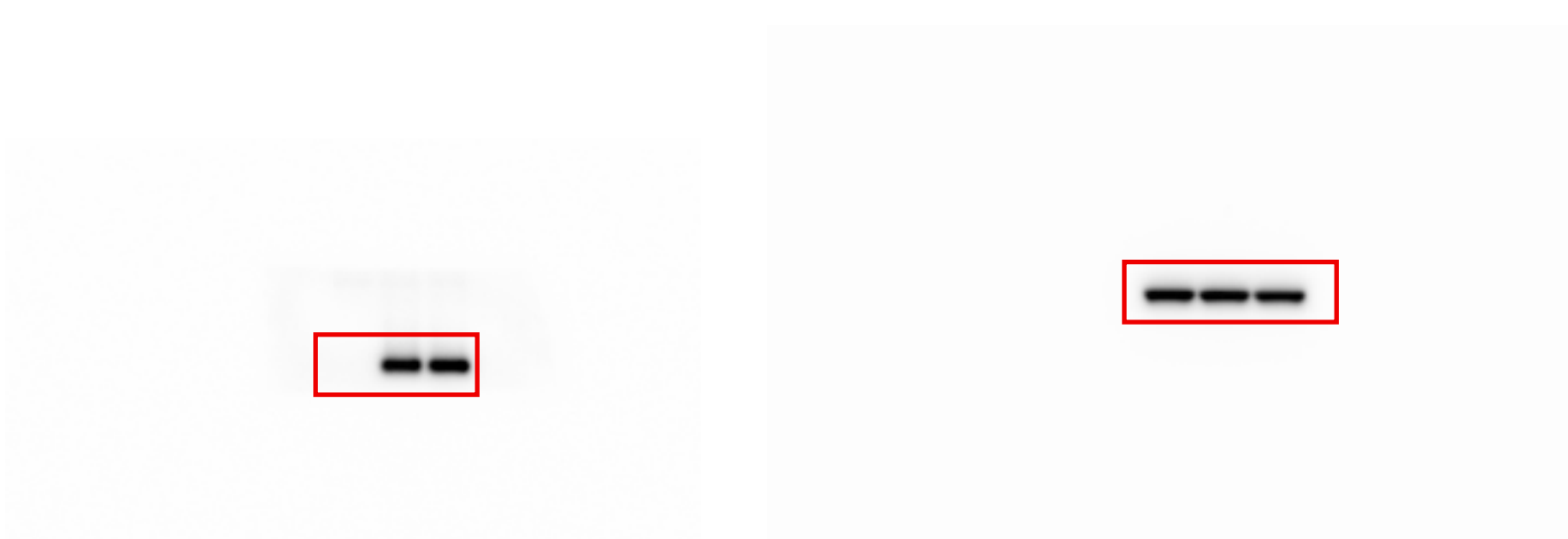

Fully unedited blot/gel for Figure 4A

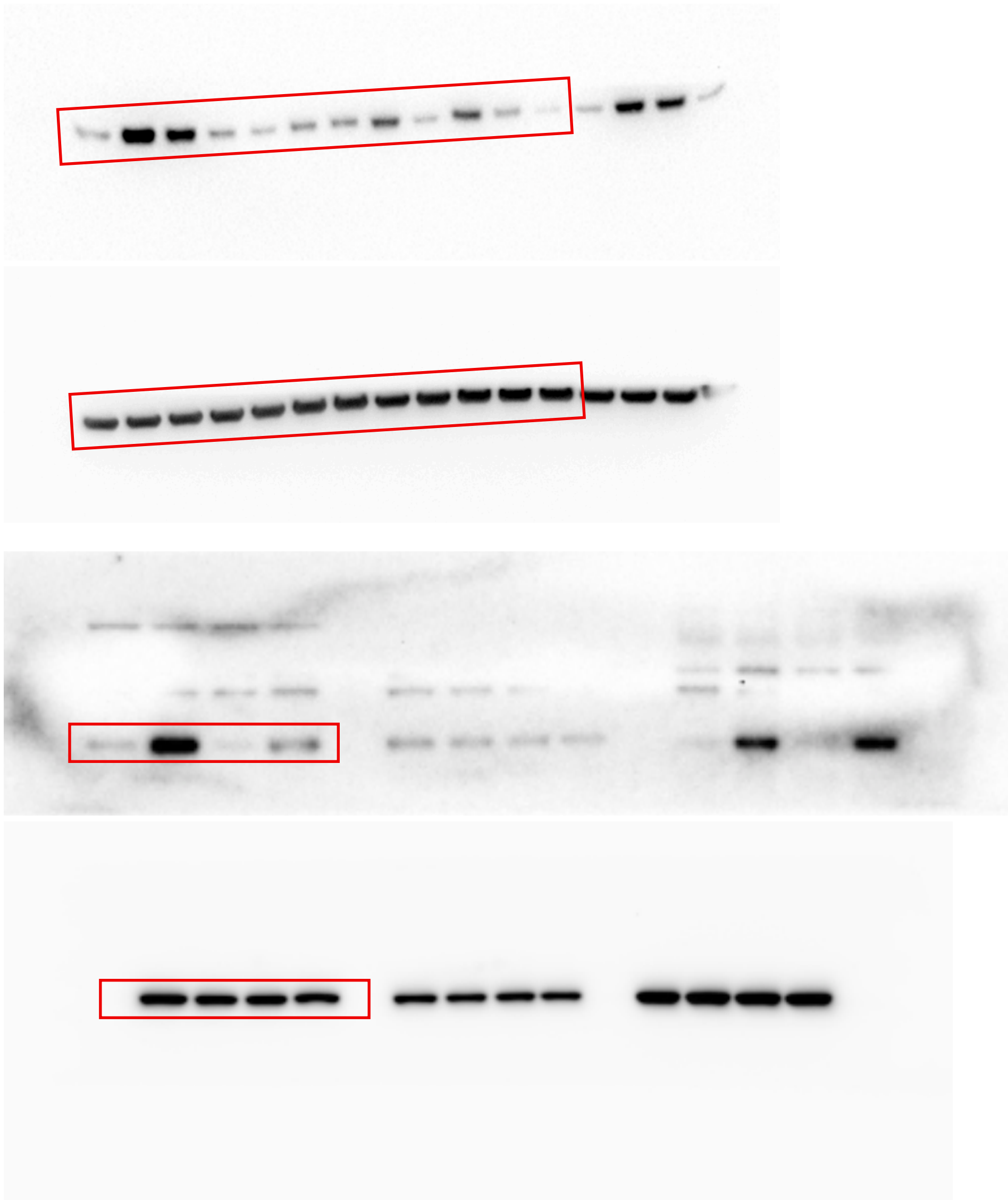

Fully unedited blot/gel for Figure 4J

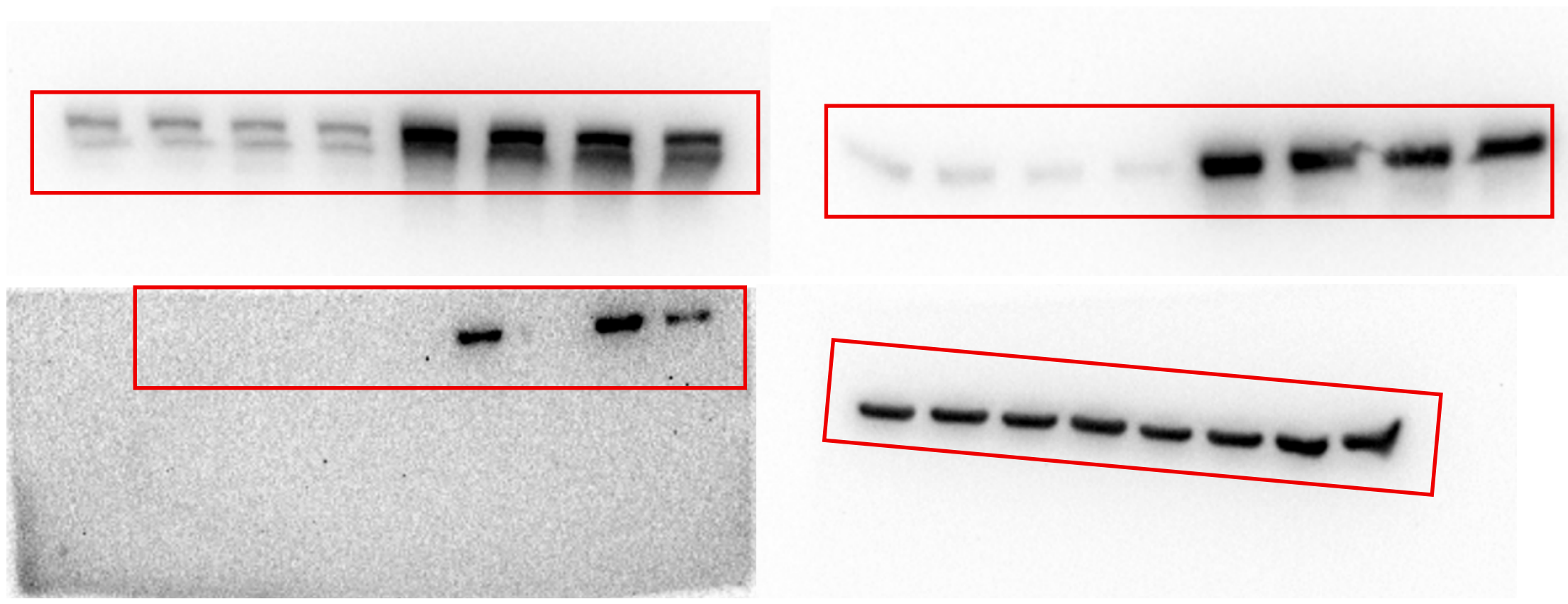

Fully unedited blot/gel for Supplemental Figure 1A

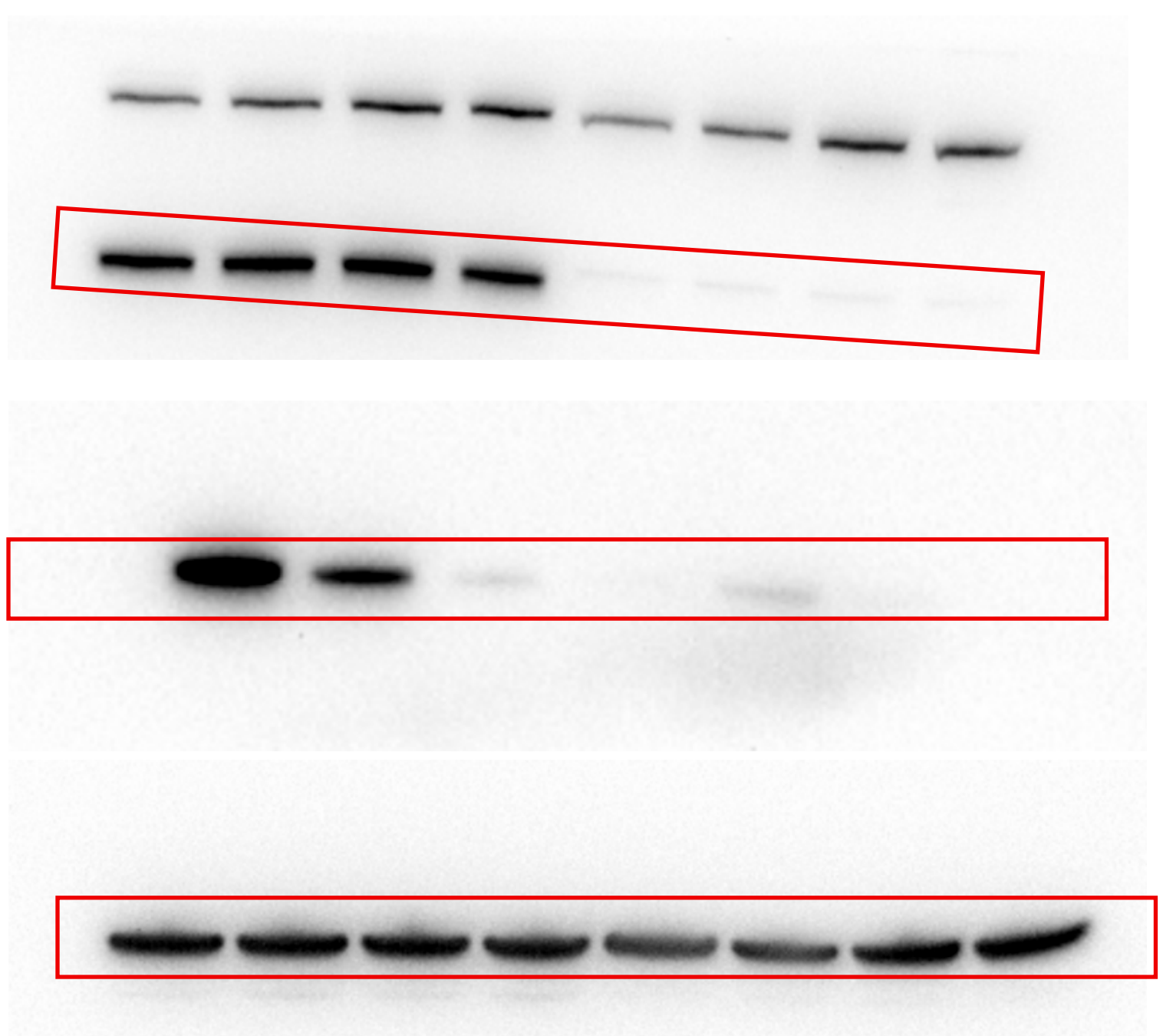

Fully unedited blot/gel for Supplemental Figure 5D

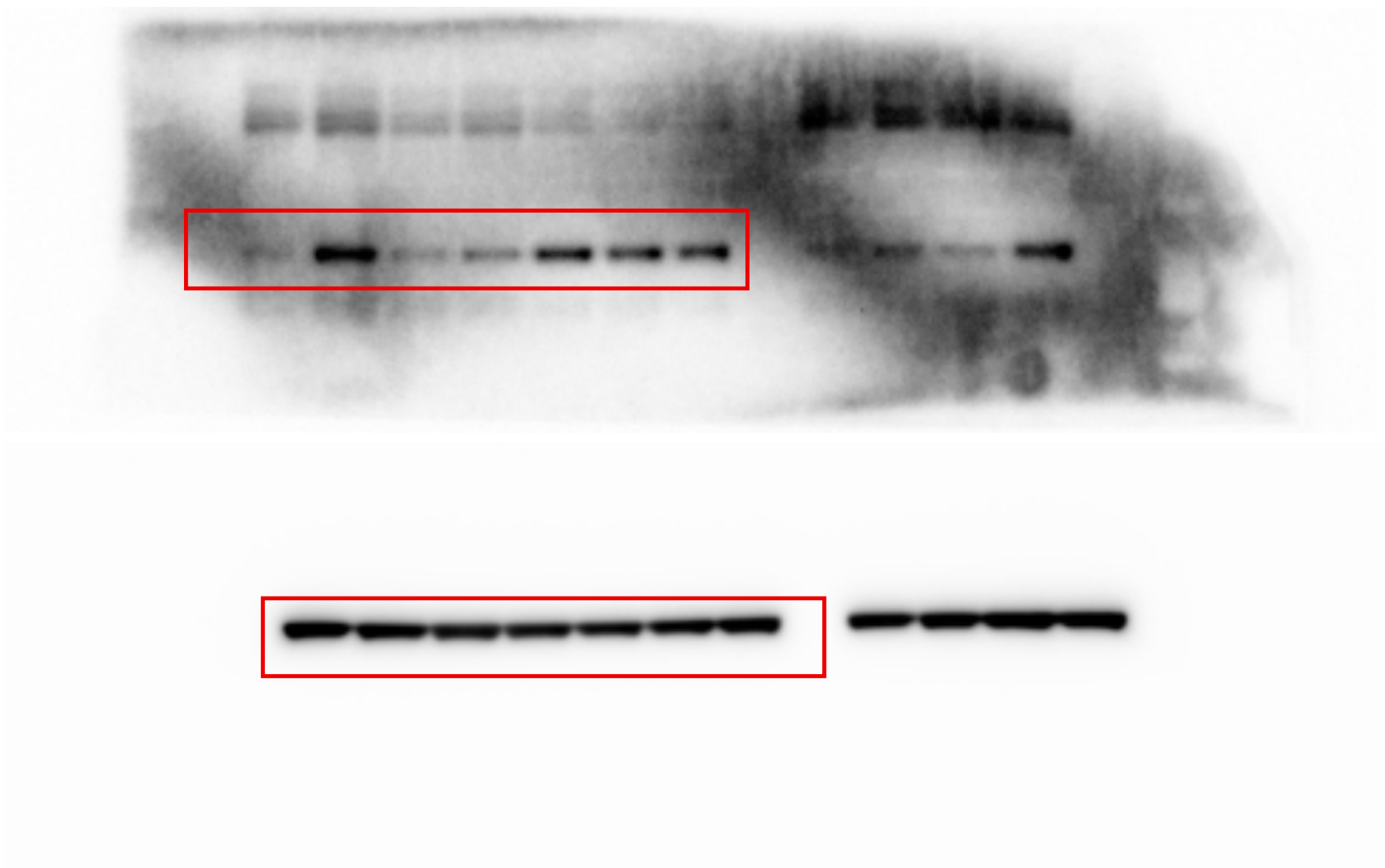

Supplement: Supplementary file 2 [file jci-136-192225-s122.pdf]
